# Sequence and tissue targeting specificity of ZFP36L2 reveals *Elavl2* as a novel target with co-regulation potential

**DOI:** 10.1093/nar/gkac209

**Published:** 2022-04-05

**Authors:** Ian C Redmon, Matthew Ardizzone, Hilal Hekimoğlu, Breanne M Hatfield, Justin M Waldern, Abhishek Dey, Stephanie A Montgomery, Alain Laederach, Silvia B V Ramos

**Affiliations:** Biochemistry and Biophysics Department, University of North Carolina, Chapel Hill, NC 27599, USA; Biochemistry and Biophysics Department, University of North Carolina, Chapel Hill, NC 27599, USA; Biochemistry and Biophysics Department, University of North Carolina, Chapel Hill, NC 27599, USA; Chemistry Department, University of North Carolina at Chapel Hill, Chapel Hill, NC 27599, USA; Biology Department, University of North Carolina, Chapel Hill, NC 27599, USA; Biology Department, University of North Carolina, Chapel Hill, NC 27599, USA; Department of Pathology and Laboratory Medicine, University of North Carolina, Chapel Hill, NC 27599, USA; Biology Department, University of North Carolina, Chapel Hill, NC 27599, USA; Bioinformatics and Computational Biology Program, University of North Carolina, Chapel Hill, NC 27599, USA; Biochemistry and Biophysics Department, University of North Carolina, Chapel Hill, NC 27599, USA

## Abstract

Zinc finger protein 36 like 2 (ZFP36L2) is an RNA-binding protein that destabilizes transcripts containing adenine-uridine rich elements (AREs). The overlap between ZFP36L2 targets in different tissues is minimal, suggesting that ZFP36L2-targeting is highly tissue specific. We developed a novel *Zfp36l2*-lacking mouse model (L2-fKO) to identify factors governing this tissue specificity. We found 549 upregulated genes in the L2-fKO spleen by RNA-seq. These upregulated genes were enriched in ARE motifs in the 3′UTRs, which suggests that they are ZFP36L2 targets, however the precise sequence requirement for targeting was not evident from motif analysis alone. We therefore used gel-shift mobility assays on 12 novel putative targets and established that ZFP36L2 requires a 7-mer (UAUUUAU) motif to bind. We observed a statistically significant enrichment of 7-mer ARE motifs in upregulated genes and determined that ZFP36L2 targets are enriched for multiple 7-mer motifs. *Elavl2* mRNA, which has three 7-mer (UAUUUAU) motifs, was also upregulated in L2-fKO spleens. Overexpression of ZFP36L2, but not a ZFP36L2(C176S) mutant, reduced *Elavl2* mRNA expression, suggesting a direct negative effect. Additionally, a reporter assay demonstrated that the ZFP36L2 effect on *Elavl2* decay is dependent on the *Elavl2-*3′UTR and requires the 7-mer AREs. Our data indicate that *Elavl2* mRNA is a novel target of ZFP36L2, specific to the spleen. Likely, ZFP36L2 combined with other RNA binding proteins, such as ELAVL2, governs tissue specificity.

## Graphical Abstract

**Graphical Abstract:**
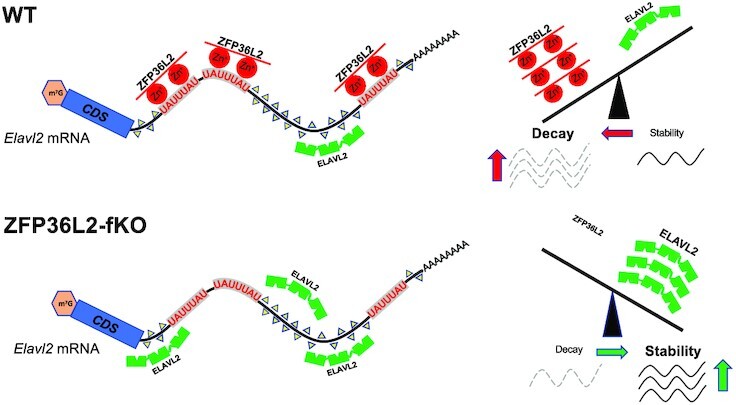
Elavl2 mRNA modulation by ZFP36L2 and ELAVL2. ZFP36L2 prevails over ELAVL2 in WT. Auto-positive feedback loop likely predominates in ZFP36L2-fKO favoring ELAVL2 effects on its own transcript.

## INTRODUCTION

RNA-binding proteins (RBPs) affect the fate of their target transcripts by modulating distinct aspects of mRNA metabolism, such as splicing, editing, localization, and stability. In addition, RBPs function as translation gatekeepers to control the final amount of protein produced at specific times and locations in cells. These properties of RBPs establish the cell biology of a tissue and, in turn, the physiology of an organism. These functional characteristics of RBPs grant them the deserved classification of ‘key regulators’ of gene expression. The human genome encodes about 424 known and predicted RBPs (1); however, only a small fraction of RBPs have been functionally validated and characterized (2–4).

A prominent class of RNA-binding proteins is the adenine-uridine-rich element (ARE) RBPs (5–7). Members of this class of RBPs either stabilize or destabilize their target transcripts. The final stability of a particular transcript can be modulated by multiple RBPs competing to bind in a simultaneous or in a mutually exclusive manner to that same transcript. A major challenge is to explain the specificity of ARE-RBP targeting and how the functions of these proteins are determined. AREs encompass many distinct nucleotide sequences rich in adenines and uridines; AREs are usually found in the 3′UTRs of mRNAs that have relatively short half-lives (8,9). The AUUUA pentamer is considered the minimum core motif. AREs are divided into several classes (10,11) based on the numbers of pentamers and their context. Intriguingly, the same ARE motif in different sequence contexts can have different effects on the final mRNA binding and/or stability (12,13).

In this study, we focus on the physiological role of ZFP36L2, Zinc finger protein like 2, also referred to as TIS11D (14), ERF2 (15), and BRF2 (16). ZFP36L2 is a member of the group ARE-binding proteins referred to as either the tris-tetraprolin (TTP) family (17) or the zinc finger protein 36 (ZFP36) family. ZFP36 proteins interact directly with transcripts that contain AREs, whereupon they recruit mRNA decay factors such as the exosome, the decapping enzyme (18), and the CCR4 deadenylase (18,19), thereby accelerating mRNA degradation. The first genetic study of the *Zfp36l2* gene in mice resulted in a surprising phenotype of ovarian female infertility due to arrest of the early embryo development at the two-cell stage (20). However, in this original mouse model the disruption of the first exon still resulted in the expression of a truncated protein, ΔN-ZFP36L2, lacking 19 amino acids at the amino-terminal end, which was also expressed at lower levels. The persistence of a truncated protein containing the functional domain, the tandem zinc finger (21), kept us from directly investigating potential mRNA targets, other than the finding of *Lhr* mRNA as a ZFP36L2 target due to its role in ovulation and oocyte maturation (22). Once a conventional knockout was created, a later developmental problem was also observed, as homozygous pups lacking any ZFP36L2 expression did not survive past the second week of life (23), likely due to severe anemia and pancytopenia. Because these animals did not survive past sexual maturation, an evaluation of the female reproductive function was not feasible. Meanwhile, we observed that oocytes exhibit 8-fold higher expression of *Zfp36l2* mRNA than macrophages (21). Recently, the female infertility phenotype was confirmed using an elegant mouse model in which *Zfp36l2* was specifically removed from the oocytes (24). Additionally, ZFP36L2 knockdown in erythrocyte lineages revealed the requirement of this protein for self-renewal and subsequent differentiation into erythroid cells (25).

Most studies of ARE specificity of the ZFP36 family have examined only the prototypical family member, ZFP36. Thus, little is known about the specificity of the other family members. Interestingly, several mRNAs whose expression was regulated by ZFP36L2 were identified in a knockdown model with erythrocyte lineage cells (25) or an oocyte knockout model of ZFP36L2 (24). In the erythrocyte knockdown model, ∼72% of 5278 potential ZFP36L2-mRNA targets contain the AUUUA motif (25). In the oocyte knockout model, 1418 mRNAs were expressed at higher levels compared with wild type oocytes. Curiously, many mRNAs that encode transcription factors appeared in both data sets, although there was only minimal overlap of the overall potential transcripts regulated by ZFP36L2 in these two sets. This observation led us to the hypothesis that ZFP36L2 targeting occurs in a tissue specific manner.

**Figure 1.**
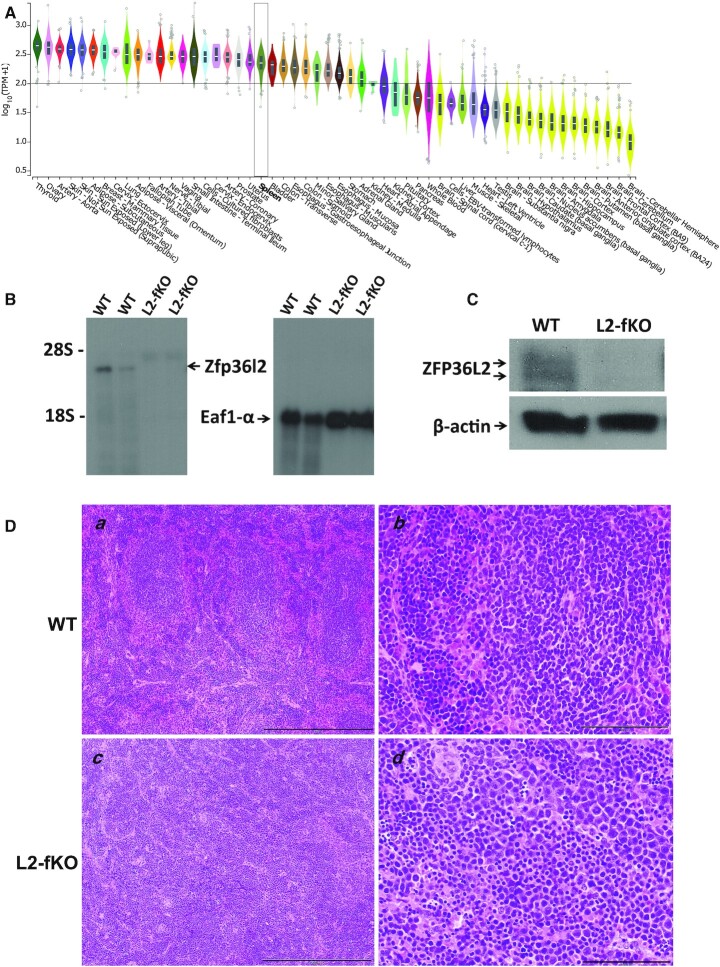
Tissue expression and visualization of *Zfp36l2* knockout in the spleen. (**A**) Tissue expression in transcripts per million (TPM) on a log scale for *Zfp36l2* mRNA from 17 382 samples sequenced by the GTEx RNA-seq consortia. Median *Zfp36l2* expression is indicated with a horizontal line and the expression in the spleen is boxed. (**B**) Total RNA was isolated from wild-type and L2-fKO mouse spleens. Five micrograms of each sample was used in Northern blots, probed with *Zfp36l2* and *Eaf1-α*^32^P labeled probes, to confirm lack of *Zfp36l2* mRNA in L2-fKO samples. The *upper band* on the left panel corresponds to *Zfp36l2* and *lower band* on the right panel to the *Eaf1-α* loading control (*arrows*). (**C**) Immunoblot analysis of spleen protein extracts from wild-type and L2-fKO mice. Twenty-five micrograms of protein was loaded per lane and probed with C2-Zfp36l2-AS rabbit polyclonal Ab (1:10 000) and mouse monoclonal $\beta$-actin-Ab (1:10,000) to confirm absence of ZFP36L2 protein in L2-fKO samples. (**D**) Representative histological samples of wild-type and L2-fKO spleens (13 and 14 days old, respectively) stained for hematoxylin-eosin (H&E) and microphotographed at 100× (***a***, ***c***) and 400× magnification (***b***, ***d***). The black line at the bottom-right of all figures corresponds to 100 mm.

To determine if this apparent tissue selectivity of ZFP36L2 is present in other tissues, we analyzed potential mRNA targets of ZFP36L2 in the spleen, using our novel CMV-*Cre* conditional knockout of *Zfp36l2*, referred to as L2-fKO mouse model. This L2-fKO model was created by crossing a CMV-*Cre* mouse line with a *Zfp36l2*^fl/fl^ mouse line. We chose to analyze the lack of ZFP36L2 in the spleen for two reasons. First, *Zfp36l2* is expressed in the spleen at moderate to high levels based on our previous findings in mice (20,21) and in the human Gene Tissue Expression atlas (Figure 1A). Second, because the expected phenotype of our L2-fKO includes anemia and pancytopenia, and under this pathological condition, the spleen is capable of extramedullary hematopoiesis. Thus, we suspected that the spleen would harbor ZFP36L2-target transcripts of physiological relevance. In addition, this analysis allows us to determine mRNA features, common or unique, that may be responsible for ZFP36L2 tissue specificity. We hypothesized that, in the absence of ZFP36L2, the abundance of some of its target mRNAs would be elevated. Thus, we performed an RNA-seq transcriptome analysis with wild type and L2-fKO spleen samples. The analysis of our differential gene expression in the spleen further confirmed that ZFP36L2 targeting is highly tissue specific. In addition, we validate *Elavl2* as a novel mRNA target of ZFP36L2.

## MATERIALS AND METHODS

### Mouse model

Conditional *Zfp36l2* knockout mice carrying the loxP sites flanking exon 2 of *Zfp36l2* (*Zfp36l2*^fl/+^) were generated by Xenogen Biosciences (Cranbury, NJ) using standard embryonic stem (ES) cell targeting techniques. Mice with both *Zfp36l2* alleles containing loxP sites were kindly provided by Dr. Blackshear by MTA and are referred to as *Zfp36l2*^fl/fl^ (24). B6.CTg(CMV-Cre)1Cgn/J (stock#006054) mice were purchased from Jackson Laboratory. Deletion of *Zfp36l2* by conditional knockout was obtained by crossing the CMV-Cre transgenic females with the *Zfp36l2* floxed males (*Zfp36l2*^fl/fl^) to generate *Zfp36l2*^fl/–^;CMV-Cre in the first generation (F1). When *Zfp36l2*^fl/–^;CMV-Cre (F1) females were mated with *Zfp36l2*^fl/–^;CMV-Cre (F1) males, animals of both sex with the recombined *Zfp36l2* alleles were generated in the second generation (*Zfp36l2*^–/–^;CMV-Cre). These animals are referred to as L2-fKO. Animals with both wild-type copies of *Zfp36l2* and the Tg.CMV-Cre were used as wild-type littermate controls for experiments (Supplementary Figure S1). Animals were housed in the Dental School vivarium of the UNC School of Medicine, Chapel Hill, in agreement with protocols approved by the Institutional Animal Care and Use Committee of the University of North Carolina (IACUC ID#21–048). Mice were genotyped by PCR. For the *Zfp36l2^flox,+ or-^* the following primers were used: F1: 5′ AGATACCAAACACTTAGGTCTCAGATGAG F2: 5′ CGACCA TTACAGGACCCAGAAA 3′ R: 5′ ACCACCACAAAGGAGGCTGAGA 3′. For the presence of the Tg.CMV-Cre, we used as primers: F: 5′ TGGGCCAGCTAAACATGCTT 3′ and R: 5′ GGTGTTATAAGCAATCCCCAGA 3′.

### Northern blot analysis

Dissected mouse tissues were freshly processed with the RNeasy kit (Qiagen) to extract total cellular RNA, according to the directions of the manufacturer. RNA samples (5–20 μg) were separated by electrophoresis in 1.2% agarose/formaldehyde gels and used for Northern blotting (22). The nylon membranes were hybridized with ^32^P-labeled *Zfp36l2* and *Eaf1-α* probes.

### Reverse transcriptase (RT) and real time RT-qPCR

Total cellular RNA was freshly extracted from mouse dissected tissues using RNeasy kit (Qiagen) and quantified using a NanoDrop instrument (Thermo Fisher). Three micrograms of total RNA samples were treated with DNase (TurboDNA-free kit, Invitrogen) for a total of 60 min. After that period, the total RNA was re-measured and 500 ng of RNA/DNase treated was used as template to synthesize cDNA with the High-Capacity cDNA archive kit (Applied Biosystems) according to manufacturer's instructions. The expression levels of each gene were normalized to the levels of GAPDH and expressed in relative abundance. To quantify *ZFP36*, *Zfp36l1*, *Zfp36l2*, *Apol11b*, *Fgf23*, *Mpl*, *Ikzf2* and *Irf8* genes, 5 ng of cDNA was combined with predesigned primer/probe sets and TaqMan Universal PCR Master Mix (Applied Biosystems) in a 10 μl final volume. To quantify the *Elavl2* gene, 10 ng of cDNA was used instead of 5 ng. For the GAPDH, 0.5 ng of cDNA was used. All reactions were performed in triplicate in 96-well plates. The differences in concentrations between samples was based on normalization with a single reference gene, using the ΔΔC_q_ method. The qPCR cycles were preceded by a ‘hold stage’ of 50°C for 2 min and 95°C for 10 min, followed by 40 cycles of: 95°C for 15 sec and 60°C for 1 min. Real-time assays were performed on an Applied Biosystems QuantStudio 6. Primers and probes for detection and quantification were obtained from Applied Biosystems; primer/probe sets conjugated to FAM-MGB used were: Mm00457144_m1, Mm01304623, Mm00492049_s1, Mm03992571_s1, Mm00445621_m1, Mm00440310_m1, Mm00496108_m1, Mm00492567_m1, Mm00516015_m1, Mm99999915_g1, Hs00270011_m1 and Hs02758991_g1.

### Cell culture, transfections and protein extracts

U2-OS cells (American Type Culture Collection) were cultured in McCoy medium with 10% fetal bovine serum, penicillin (100 units/ml), and streptomycin (100 μg/ml). HEK 293 cells (American Type Culture Collection) were maintained in minimum essential medium supplemented with 10% fetal bovine serum, penicillin (100 units/ml), and streptomycin (100 μg/ml). Transient transfection of 2 × 10^6^ cells seeded in 100 mm plate with different *Zfp36l2* plasmids (12,22) was performed using Lipofectamine 2000 (Life Technologies) according to the manufacturer's protocol (22). The transfection mixture was incubated with the cells for 20 h and then replaced with medium for a further 24-h incubation, after which the cells were lysed for RNA or protein extraction.

### RNA Electrophoretic mobility assay

Wild type ZFP36L2 (WT-L2) or ZFP36L2-Mut (L2-C175S), or empty vector plasmids were transiently transfected into HEK 293T cells. Then, protein extracts were prepared as described (12). The protein extracts were incubated for 15 min at room temperature with 0.2 × 10^5^ cpm of ^32^P-labeled RNA probe in a final volume of 20 μl containing 10 mM HEPES (pH 7.6), 40 mM KCl, 3 mM MgCl_2_, 0.5 μg/μl heparin, and 1.2 μg yeast tRNA, as described (12). The resultant reaction mixtures were applied to 6% nondenaturing acrylamide (37.5:1) gels and subjected to electrophoresis at 150 V for 15 min followed by electrophoresis at 200 V for 90 min in 0.4× Tris–borate/EDTA running buffer. The gels were dried, exposed to film (Carestream BIOMAX MR Film), and developed after 12–20 h of exposure.

### Preparation of RNA probes for RNA electrophoretic mobility assay

The RNA probes were synthesized with the Riboprobe System-T7 (Promega) using DNA primer sequences immediately downstream from a T7 promoter, as previously described (26). The RNA probes were body-labeled during the transcription process, which was performed in the presence of [α-32P] UTP (3000 Ci/mmol; PerkinElmer). The probes were designed to be around 30 nucleotides considering the location of the ARE motif, except for the *Tnf*-α probe, which was 58 nucleotides long. The synthesized RNA probes were separated from the free nucleotides using Sephadex G50 columns (GE Healthcare Life Sciences) and subsequently electrophoresed on a 16% polyacrylamide urea gel. The probes were purified from excised gel fragments after detection by autoradiography, as previously described (12). The amount of RNA probe used in each lane of the EMSA was calculated to be ∼10 femtomoles. The sequence of the RNA probes used are listed below. Note that the three *Ikzf2* probes were previously used in (27) and the last two probes, *Gm-csf* and *Tnf-α* were used as a positive control:

*Ikzf2* ARE1: 5′UUUACUAGGGCUAUUUAUUCCACUAUUU
*Ikzf2* ARE2: 5′AAGGAUAUUUAUUUCUGAAUGAGGUAAAUAAGUU
*Ikzf2* ARE3: 5′UUAUUCAUAUUUAUAUGUAGUGUGUUCU
*Mpl*: 5′GUGGGGUCUUGCUGUCAUCUAUUUAUUUGAUCUCUC
*Mpl* mut: 5′GUGGGGUCUUGCUGUCAUAUAUUUAUUUGAUCUCUC
*Irf8*: 5′AAGGAGUGCUAGUGUCCAAAUAUUUAUUUUUGUAUUCUCU
*Nfix*: 5′UGGGCUUUAUUUAUUGAGAAUCUAGUU
*Elavl2* ARE1: 5′UACAUGUAUAUAUUUAAAAAAAAAAAUAAGG
*Elavl2* ARE2: 5′UGUUGUCCUGAGGACUUGAAUUUACAGUGCAUCA
*Elavl2* ARE3: 5′UGAGCUCUUGUCCUCAGUCCAUUUAUAUAUGA
*Elavl2* ARE4: 5′UGUAAGGCUGGUAUUUAUUUGAAGUUGUACA
*Elavl2* ARE5: 5′CAAACAGUAUUUAUUUUGUAAUUCUGAUUUG
*Elavl2* ARE6: 5′UUUGAAGUUUACAUUUUUAUUUAUGAAGUUACAAA
*Apol11b*: 5′AAUGUCAGAAAUAUUUAUUUUCUUGAAGA
*Fgf23*: 5′GUUAAUCUGAUUUAAAGACCCCAACAGGUAAAC
*Gm-csf*: 5′UUUAUUUAUUUAUUUAAGUUCAUAUUCCAU
*Tnf*-alpha: 5′UGAUUAUUUAUUAUUUAUUUAUUAUUUAUUUAUUUACAGAUGAAUGUAUUUAUUUGGG

### Construction of nanoluciferase *Elavl2* 3′UTR reporter system

The full length 3′UTR sequence of *Elavl2* including the endogenous poly(A) site corresponding to 2529 bp of NM_001374696.1 was gene synthesized (Genewiz) and is referred as WT-3′UTR *Elavl2*. Using NEB Q5 Site directed mutagenesis kit and the gene synthesized as a template, the three 7-mer AREs from *Elavl2* 3′UTR were deleted to create ΔARE 3′UTR *Elavl2* (2508 nt long). The WT-3′UTR *Elavl2* and the ΔARE 3′UTR *Elavl2* sequences, which differ in size by 21 nt were cloned into pNL3.2.CMV (Promega) using XhoI and NheI restriction sites located downstream of the PEST domain. The pNLD3.2.CMV vector expresses nanoluciferase in frame with a PEST domain that prevents protein accumulation ensuring a short half-life of nanoluciferase. All constructs were verified by Sanger sequencing and restriction digest.

### Luciferase experiments

HEK 293 cells were plated in a six-well plate (∼100 000 cells/ml) and transfected the next day. When double transfections were performed, 550 ng of DNA was transfected: 500 ng of control firefly luciferase (pGL4.5 vector) and 50 ng of nanoluciferase constructs (pNL3.2.CMV vector) were used with standard quantities of Lipofectamine 2000 in serum-free Optimem (Fisher). When triple transfections were performed, 500 ng of control firefly luciferase (pGL4.5 vector), 50 ng of nanoluciferase constructs (pNL3.2.CMV vector) and 5 ng of ZFP36L2 or ZFP36L2-C176S mutant, totaling 555 ng of DNA was transfected. After 24 hours, protein extracts were prepared to measure luminescence using the Nano-Glo Dual-Luciferase Reporter Assay System (Promega). Each sample had its luminescence measured in triplicate on the CLARIOstar (BMG Labtech) plate reader. Three biological replicates from separate days were combined for analysis.

### RNA sequencing and data analysis

An Illumina HiSeq by Genewiz (https://www.genewiz.com/) produced 461 851 481 reads. On average, 50–60 million reads per sample (4 wild type and 4 L2-fKO) were trimmed by Trimmomatic v.0.36 to remove possible adapter sequences and nucleotides with poor quality sequence. In all cases, over 97.5% of the trimmed reads were mapped with STAR aligner v.2.5.2b (28) to the *Mus musculus* ENSEMBL GRCm38 reference genome. STAR aligner is a splice aligner that detects splice junctions and incorporates them to aid aligning the entire read sequences. Unique gene hit counts were calculated by featureCounts from the Subread package v.1.5.2. Only unique reads that fell within exon regions were counted in a strand-specific manner because the library was strand-specific.

After extraction of gene hit counts, the gene hit counts table was used for downstream differential expression analysis. Using DESeq2, we performed a comparison of gene expression between the wild-type and L2-fKO samples (29). The Wald test was used to generate *P*-values and log2 fold changes. Genes with an adjusted *P*-value <0.05 and absolute log_2_ fold change >1 were called as differentially expressed genes for each comparison.

We obtained 3′UTR sequences for all genes detected with at least 10 reads in the wild-type and L2-fKO samples, and the longest sequence was used for subsequent motif analysis. The ARE*score* program (30) was used to compute the AREscores for all these 3′UTRs and then analyzed for the upregulated, downregulated and unchanged categories (30). Seven-mer ARE and GAAA motifs were counted using custom Matlab (https://www.mathworks.com) scripts. Kolmogorov–Smirnov tests, Venn overlap analysis, and graphs were performed using both R-project for statistical computing (https://www.r-project.org) and Prism (https://www.graphpad.com/scientific-software/prism/).

## RESULTS

### Tissue expression of *Zfp36l2* mRNA in humans and mice

We assessed *Zfp36l2* expression in different tissues by visualizing median levels of the *Zfp36l2* mRNA in samples from individuals sequenced by the Gene Tissue Expression (GTEx) atlas (31). We identified 30 tissues in which the median expression was above 100 transcripts per million (TPM); thus, the *Zfp36l2* gene is highly expressed in most tissues (Figure 1A). The tissue of interest for this study was the spleen, which had a median *Zfp36l2* mRNA expression of 249 TPM, a moderately high expression. In mice, we also found that *Zfp36l2* mRNA was highly expressed in the spleen (20,21).

To determine the effect of the absence of ZFP36L2 in the splenic transcriptome, we created a CMV-*Cre* conditional knockout mouse model (L2-fKO) of *Zfp36l2*. We crossed a CMV-*Cre* mouse with another mouse line, *Zfp36l2*^fl/fl^ in which the *Zfp36l2* gene was flanked by *lox-P* sequences. In the CMV-*Cre* mouse, the *Cre* gene is under the transcriptional control of a strong promoter, the human cytomegalovirus minimal promoter (CMV), which is active during early embryogenesis and leads to Cre enzyme expression in all tissues, including germ cells. By crossing these two mouse lines, we achieved 100% recombination of the *Zfp36l2* gene flanked by *lox-P* sequences and deletion of *Zfp36l2* gene in all tissues of the L2-fKO mouse. The deletion of *Zfp36l2* was confirmed by PCR assays of multiple tissues such as the liver, spleen, thymus, ovary, and kidney (not shown).

We used Northern blotting to confirm the absence of *Zfp36l2* mRNA in the L2-fKO spleen (Figure 1B, left panel); we probed for the *Eaf1-*$\alpha$ gene as a loading control (Figure 1B, right panel). We verified the absence of the ZFP36L2 protein in L2-fKO spleen by immunoblotting, with $\beta$-actin as a control (Figure 1C). Northern blot analysis (Figure 1B) confirms that *Zfp36l2* mRNA is expressed as a single transcript isoform, whereas two bands are observed in the Western blot (Figure 1C). This is consistent with previous studies (22,32) showing a similar migration pattern corresponding to a highly phosphorylated protein; thus, the two bands likely correspond to different phosphorylation states. All L2-fKO pups died before the second week of life; however, one L2-fKO female survived 14 days. Our model resulted in a similar phenotype as the previously reported conventional KO mice (23). As in this previous KO model, we observed anemia and pancytopenia (Supplementary Figure S2). Extramedullary hematopoiesis (hematopoiesis in organs outside of the bone marrow) is a compensatory mechanism to overcome the inefficiency of the bone marrow in producing red blood cells and white blood cells. This physiological compensatory mechanism is usually triggered during fetal development in the liver and in the spleen. In the conventional KO model, Stumpo *et al.* studied the fetal liver, which is normally a site of extramedullary hematopoiesis during fetal development (23). After birth, only the bone marrow is expected to remain the major site of hematopoiesis; however, extramedullary hematopoiesis can occur under pathological conditions. The severe pancytopenia in conjunction with a developmental problem are a compound reason for pathological extramedullary hematopoiesis. Thus, we asked whether the spleen would harbor compensatory hematopoiesis after birth in the L2-fKO animals. We performed a histological analysis of spleen sections stained with hematoxylin-eosin (H&E) as shown in Figure 1D panels *a* to *d*. Wild-type spleen displayed the expected white pulp and red pulp organization (*a*), with lymphoid follicles and surrounding mantle/marginal zones. However, the L2-fKO spleen contained large nodules of disorganized white pulp (*c*). Higher magnification revealed that these ‘*mega*’ nodules were composed of a population of pleomorphic mononuclear cells, characterized by large and small sized cells (*d*). All L2-fKO pups from which we collected tissues (*n* = 32), from day 5 to day 10, presented these macroscopic splenic pathognomonic abnormalities (Supplementary Figure S3).

### Differential gene expression profile of wild type and L2-fKO spleen

**Figure 2.**
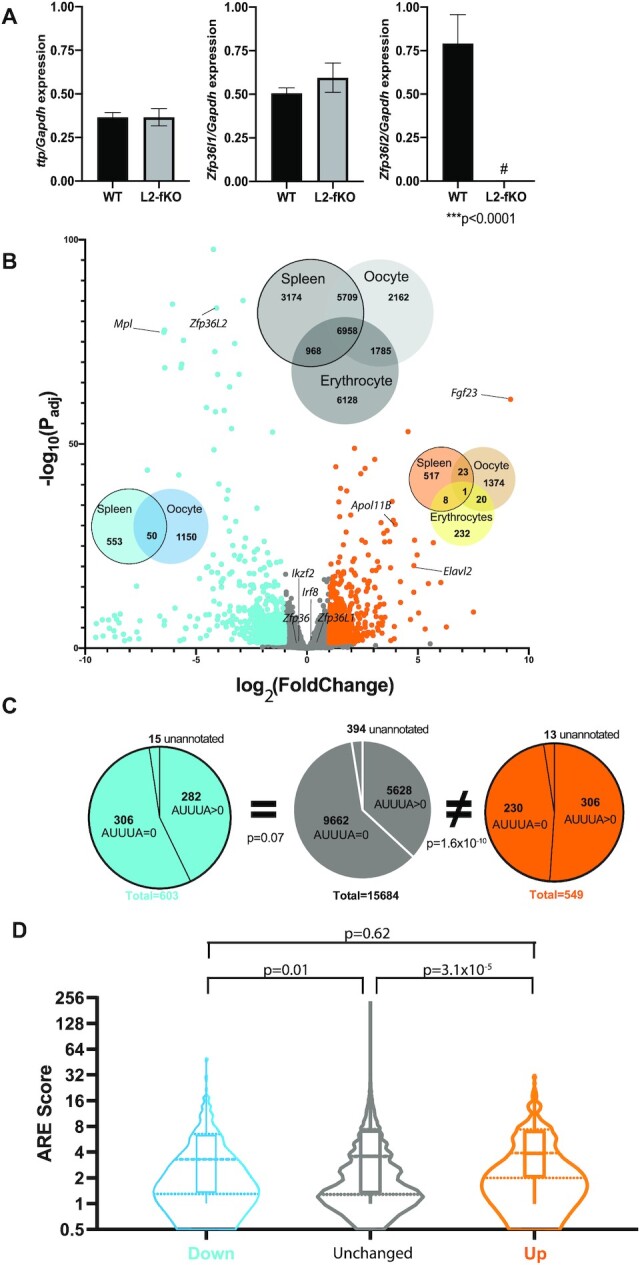
Molecular characterization of gene expression in the L2-fKO spleen. (**A**) Expression levels of *Zfp36* family members were normalized to *Gapdh* and expressed in relative abundance. *Zpf36l2* was barely detectable in the L2-fKO spleen samples (right panel). The mRNA levels of the other family members, *Zfp36* (left panel) and *Zfp36l1* (central panel) were not significantly different between wild type (*n* = 11) and L2-fKO (*n* = 12) samples (values are mean ± SEM; *P* values were calculated using Student's *t*-test). (**B**) Volcano plot of differential gene expression of wild type (*n* = 4) versus *Zfp36l2* (*n* = 4) knockout spleens, with cut off |log_2_FC > 1| and adjusted *P*-value <0.05. The 549 upregulated genes are in orange, and the 603 downregulated genes are blue. The remaining 15 684 genes that were not differentially expressed genes are plotted in gray. RT-qPCR validation was performed for the genes specified in the volcano plot (Supplementary Figure S4). The gray Venn diagram illustrates all genes expressed in spleen, erythrocytes lineage, and oocytes detectable by RNA-seq, microarray and scRNA-seq, respectively. Venn diagrams in blue and orange represent genes differentially down- and upregulated, respectively, in all three tissues. (**C**) Occurrence of 5-mer (AUUUA) AREs in the 3′UTR of genes detected in the spleen. Left, middle and right pie chart corresponds to downregulated (cyan), unchanged (gray) and upregulated (orange) genes in spleen samples, respectively. The number of genes lacking (AUUUA = 0) or containing one or more 5-mer (AUUUA > 0) are represented in each respective pie chart. *P* values were calculated using a Kolmogorov–Smirnov test. (**D**) Violin plots of ARE*Score* algorithm in down- (blue), unchanged (gray), and upregulated genes (orange). The rectangle in the center of the plots represents median and 25 and 75 percentiles for each plot. *P* values were calculated using a Kolmogorov–Smirnov test.

Because the three ZFP36 family member proteins bind *in vitro* to the same ARE class II probe (22,33), we were curious to learn whether, in the absence of *Zfp36l2*, the other two family members would increase to compensate for the absence of *Zfp36l2*. Thus, we used RT-qPCR to measure the amount of mRNA of *Ttp (Zfp36*), *Zfp36l1*, and *Zfp36l2* in the spleen of L2-fKO mice (Figure 2A). Both *Zfp36* and *Zfp36l1* expression levels were similar in wild type and L2-fKO samples, which suggests an absence of a compensatory mechanism from the other family members. As such, our L2-fKO model is likely a good system to identify novel ZFP36L2-targets.

Based on the well-established role of ZFP36L2 in ARE-mediated mRNA degradation, it is reasonable to hypothesize that some ZFP36L2-target mRNAs would increase in abundance in the absence of ZFP36L2. To identify mRNAs dysregulated due to the *Zfp36l2* conditional deletion, we prepared a poly(A) library from total RNA from wild-type and L2-fKO spleens from four age-matched pairs of animals. The libraries were subjected to RNA-seq analysis. Using the criteria of |log_2_FC|>1 and an adjusted *P*-value <0.05, we found 549 upregulated (Figure 2B, orange circle and dots) and 603 downregulated mRNAs (Figure 2B, cyan circle and dots) in ZFP36L2-lacking cells relative to wild type. We also detected 15 684 expressed genes whose expression was not significantly different between wild-type and L2-fKO spleens (hereafter referred to as unchanged; Figure 2B, spleen gray circle and dots). We then performed RT-qPCR validation of selected mRNAs from each category. Three genes, *Apol11b*, *Elavl2* and *Fgf23*, which were found to be upregulated by RNA-seq analysis, were also found to be significantly increased in the RT-qPCR analysis (Supplementary Figure S4). *Zfp36l2* (Figure 2A) and *Mlp* (Supplementary Figure S4) mRNAs, found by RNA-seq analysis to be downregulated, were also decreased in L2-fKO spleens by RT-qPCR analysis. Interestingly, we found that *Ikzf2* and *Irf8* mRNAs, which are modulated by ZFP36L2 in T cells (27), were located in the unchanged expression category in the spleen by the RNA-seq analysis (Figure 2B, gray). While this was confirmed for *Irf8* transcript by RT-qPCR, the *Ikzf2* mRNA level was modestly, yet significantly, decreased in the L2-fKO spleen in comparison to wild type (Supplementary Figure S4). Note also that *Zfp36* (*Ttp*) and *Zfp36l1* expression fall in the gray area of unchanged genes in the volcano plot (Figure 2B), consistent with our RT-qPCR analysis.

To further characterize the upregulated genes found in our RNA-seq data, we performed gene ontology (GO) enrichment analysis and found multiple terms are enriched, with up to 4.7-fold enrichment and with at FDR < 10^–6^ (Supplementary Table S1). The most statistically significant terms are related to the response to stimulus and extracellular matrix, processes which are known to be related to mRNA metabolism.

We next compared gene expression in L2-fKO spleens with two published analyses of ZFP36L2-KD in murine erythrocyte lineage cells (25) and ZFP36L2-KO in oocytes (24). We also attempted to identify differentially regulated genes in the original conventional knockout published in 2009 (fetal liver microarray analysis) (23); however, there is no available list of differentially expressed genes from this model and we were not able to quantitatively reproduce the number of genes expected from a re-analysis of the raw the Gene Expression Omnibus accession data. The comparison of genes differentially expressed in the spleen, erythrocyte, and oocytes is represented in the Venn diagrams of Figure 2B. Although we observed considerable overlap in the number of genes expressed in these three tissues (gray Venn diagram), unexpectedly, we observed little overlap in the upregulated genes when ZFP36L2 was absent in these tissues (orange, yellow, and cream Venn diagrams). The only upregulated gene in all three tissues was *Mthfr* (methylenetetrahydrofolate reductase), which does not contain an ARE and is likely increased due to an indirect effect. This little overlap in the upregulated genes in the three different tissues suggests that the ZFP36L2-targets vary according to the tissue evaluated. We thus hypothesized that ZFP36L2 controls different mRNA pools depending on the organ, tissue, or cell evaluated. One limitation of these comparisons is that previously published data were obtained using different techniques (microarray versus scRNA-seq) and were derived from different biological models (*ex vivo* knockdown vs *in vivo* knockout). We therefore extended our testing to another organ, the ovary, using the same biological model and RNA-seq technique. We chose the ovary because it is the organ with the second highest *Zfp36l2* expression (Figure 1A) and this gene has been implicated in mouse female infertility (12,20,22,24). Interestingly, in our preliminary histological evaluation of the neonatal L2-fKO ovary, we did not observe any major abnormality (not shown). However, in humans, *Zfp36l2* seems to be relevant after puberty as it is involved in polycystic ovarian syndrome (34). We collected ovaries from wild-type (*n* = 3) and L2-fKO (*n* = 3) age-matched animals and prepared poly(A) libraries from total RNA. We analyzed the RNA-seq data using the criteria of |log_2_FC|>1 and the adjusted *P*-value <0.05. When we compared upregulated genes in the ovary and spleen of the L2-fKO versus wild-type animals, we observed few common genes (Supplementary Figure S5), consistent with what we observed in Figure 2B (orange, yellow and cream Venn diagram). Only 13 genes are simultaneously upregulated in the spleen and ovary in our data, even though both organs share >14 792 genes (Supplementary Figure S5). This result suggests that ZFP36L2-targeting depends on the tissue analyzed and is likely modulated by factors other than the presence of AU rich elements in the 3′ UTR.

We (12,21,22) and others (35,36) have shown that ZFP36L2 is an RNA-binding protein that recognizes adenine-uridine-rich elements (ARE) located at the 3′UTRs of target mRNAs. Binding leads to mRNA destabilization (27) or inhibition of translation (37). Because ARE pentameric sequences, AUUUA, are enriched in transcripts subject to decreased stability by the ZFP36 protein family, we decided to measure the occurrence of the AUUUAs in the three categories, downregulated, unchanged, and upregulated genes from our RNA-seq results. Note that some transcripts had poor genomic annotations and were excluded as ‘unannotated’ because we could not accurately identify their 3′UTRs.

We found a significant increase in the number of 5-mer ARE motifs in the 3′UTRs of upregulated genes (*P* = 1.6 × 10^–10^) in L2-fKO spleen in comparison to unchanged genes (Figure 2C). In contrast, we did not find an increased occurrence of 5-mer AREs in the downregulated genes (*P* = 0.07). Interestingly, further analysis of the downregulated genes revealed that this slight increase in 5-mer ARE motifs in the downregulated genes is likely due to the decreased number of cells from the hematological lineage expected to be present in the spleen from the L2-fKO mice. Particularly, cells from the erythropoietic lineage expected to be found in the red pulp were reduced in the L2-fKO spleen (Figure 1D and Supplementary Figure S2). Indeed, when we subdivided the downregulated gene list found in the L2-fKO spleen into genes expressed in both the erythrocyte lineage cells and spleen; and genes expressed in the spleen but not present in erythrocyte linage cells, we observed a higher fraction of ARE 5-mers in the genes present in both samples (Supplementary Figure S6A). Thus, although some potential ZFP36L2-targets expressed only in the erythrocyte lineage (erythrocyte lineage-specific genes) show up in our analyses as downregulated genes, in fact the cells expressing these genes are absent.

**Figure 3.**
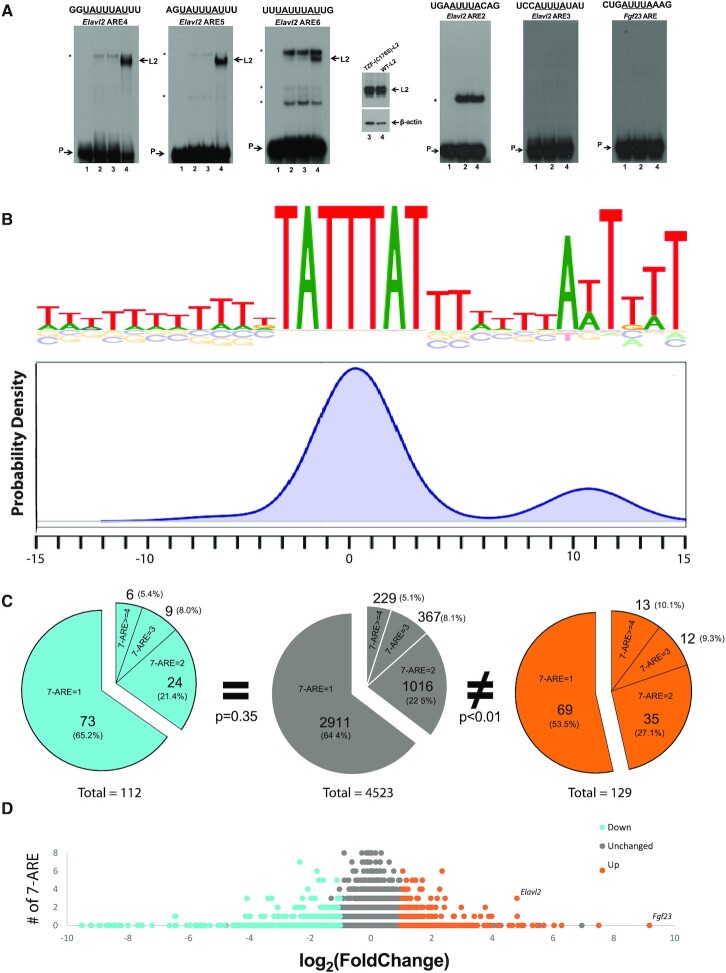
Biochemical characterization of ZFP36L2 RNA-binding specificity. (**A**) Representative RNA gel shift assays of ZFP36L2 bound to ∼30 nt long probes representing mRNAs containing the 7-mer (UAUUUAU), left panel or the 5-mer (AUUUA) ARE types, right panel. The assays were performed by incubating 0.2 × 10^5^ cpm of different ARE probes with protein extracts from HEK 293 cells transfected with a vector that expressed the ZFP36L2-C176S RNA-binding deficient mutant (20 μg, lanes 3) or with a vector that expressed wild type ZFP36L2 (20 μg, lanes 4). Immunoblotting (insert) showed that both proteins were expressed at similar levels. The migration of the probe in the absence of protein is shown in lanes 1. Note that incubation of protein extracts containing the ZFP36L2 RNA-binding mutant (lanes 3) produced similar bands as protein extracts from HEK 293 cells transfected with an empty vector (lanes 2) and are indicated by asterisks. These bands are likely endogenous proteins present in HEK cells that also interact with the probes. Complexes containing ZFP36L2 and 30 nt long probes containing a single ARE 7-mer are indicated by arrows (L2). None of the single 5-mer ARE probes bound to ZFP36L2 (lane 4, right panel). At the top of each panel, the sequence of the 7-mer or 5-mer ARE present in each probe is underlined. (**B**) Sequence logo and probability density of ‘de novo’ motif discovery using BaMMotif on the sequences of the 3′UTRs from the 127 upregulated genes in the spleen that contained one or more 7-mer AREs. (**C**) Pie charts represent the number of 7-mer ARE motifs in 3′UTRs of down- (cyan), unchanged (gray), and upregulated (orange) genes. The distributions were compared by the Kolmogorov–Smirnov test and *P* < 0.05 was considered significantly different. (**D**) Visualization of the distribution of 7-mers in the 3′UTRs of down- (blue), unchanged (gray) and upregulated (orange) genes in the *Zfp36l2* conditional knockout mouse spleen versus log_2_ of Fold Change measured by RNA-seq. *Elavl2* gene is marked among other genes significantly upregulated (orange dots).

To better understand the ARE enrichment in our RNA-seq results, we used the ARE*Score* algorithm (30) which takes into account three typical ARE features: (i) the number of AUUUA sequences, (ii) the proximity between pentamers and (iii) the presence of high AU content in the vicinity of AUUUA pentamers. ARE*Score* analysis of differentially expressed genes in L2-fKO spleen showed a statistically significant increased ARE*Score* mean for upregulated genes (*P* = 3.1 × 10^–5^) compared with unchanged genes (Figure 3D). However, a similar albeit less significant (*P* = 0.01) increase was observed in the downregulated genes. Again, a similar analysis of the ARE*Score* for those genes downregulated and expressed only in the erythrocyte lineage revealed a higher ARE*Score* than other genes downregulated in the L2-fKO spleen but not present in the erythrocyte lineage (Supplementary Figure S6B).

### Common features of ZFP36L2 functional binding sites

The minimal ARE consensus sequence is the pentamer (10,11) and the more of these motifs that are present in a given 3′ UTR, the higher the likelihood the mRNA would be a target for ZFP36 family members (38). Based on this generally accepted idea, Spasic *et al.* devised the ARE*Score* algorithm to identify transcripts enriched in ARE elements on a genome wide scale. However, the extensive validation of this approach and its biochemical testing were based on genes regulated by ZFP36, and not ZFP36L2. ZFP36L2 binds to two classic ZFP36-targets (*Gm-csf* and *Tnf-α*) in a dose dependent manner with similar affinity (Supplementary Figure S7). However, it is likely that each ZFP36 family member has different targeting specificities. Thus, it is possible that some ZFP36L2-targets may not necessarily be a specific ZFP36-target.

To identify and characterize the ZFP36L2-binding sequence, we designed 12 new RNA oligomers of about 30 nucleotides (see Material and Methods for probe's sequence), each containing a single ARE, and tested ZFP36L2 binding in an RNA electrophoretic mobility shift assay. ZFP36L2 binding appears to require a minimum 7-mer sequence (UAUUUAU) (Figure 3A, left and Supplementary Figure S8); whereas a 5-mer containing probe did not bind to ZFP36L2 (Figure 3A, right). Interestingly, probes containing a single 7-mer ARE did not bind to ZFP36 (Supplementary Figure S9), even when ZFP36 protein is expressed at a much higher levels and actively binds to a positive control. Although our gel-shift shift assays in Supplementary Figure S9 suggest that ZFP36 does not bind to a single 7-mer ARE, we cannot unequivocally exclude binding based on this assay alone, as its binding affinity maybe below the detection limit of gel shift assays.

In an attempt to identify common features of ZFP36L2, we combined the results from these 12 new RNA probes, plus 10 probes that we tested previously (12,22,27) and 5 probes assayed by other investigators (35,36,39,40) to identify 16 ZFP36L2-binding sequences from a total of 27 probes that were tested and demonstrated binding (Supplementary Table S2). In a previous study, we quantified the relative affinity of ZFP36L2 for ARE containing RNAs (12). Note that three tested probes contained the same flanking nucleotides around the 7-mer (Supplementary Table S2, *); however, they differ in almost all other nucleotides composing the remaining sequence of these probes. Particularly for *hLhr* and *mLhr* probes, even when they contained the exact same sequence binding motif, they demonstrated different binding affinities (12). Overall, this analysis supports that the minimal binding motif for ZFP36L2 is a 7-mer (UAUUUAU). In contrast, the presence of the 5-mer seems to be insufficient for binding (Figure 3A, right panel).

We performed a ‘*de novo*’ motif analysis using BaMMotif (41) on the 3′UTRs of upregulated genes in the spleen. Of the 549 genes that were upregulated in the spleen, we excluded 13 genes that were poorly annotated, which we could not accurately assign a 3′UTR. Of the remaining 536 mRNAs that were upregulated, 127 contained one or more AREs of the 7-mer type. We identified their longest 3′UTR isoform, extracted ∼30 nucleotides around the 7-mer motif seed and analyzed these sequences. We show the results of this analysis in the motif logo and probability density in Figure 3B. As a control, we used the 3′UTR sequences from the genes that were unchanged in our differential gene analysis but also contained a 7-mer motif. We did not observe any new motifs other than ARE 7-mers with strong statistical significance (*P* < 0.001). However, we did observe a uridine enrichment flanking the 7-mer ARE motifs (Figure 3B). In addition, we observe a smaller probability density peak for a second 7-mer ARE motif approximately 11 nt downstream of the seed 7-mer ARE. We observed a very similar motif and probability density in the upregulated genes in the mouse L2-fKO ovary when we performed BaMMotif analysis on their 3′UTRs (Supplementary Figure S5). This analysis suggests that ZFP36L2 preferentially binds to a single ARE 7-mer flanked by a track of uridines. This contrasts with ZFP36, which has been shown to preferentially bind to clusters of AREs of class II (17,33).

The number of 7-mer ARE motifs in 3′UTRs of downregulated (cyan) and unchanged (gray) genes had similar distributions (Figure 3C). In contrast, a comparison of the distribution between upregulated (orange) and unchanged (gray) genes was significantly different (*P* < 0.05). Additionally, we observed significant ARE 7-mer enrichment in the upregulated genes in the ovary (Supplementary Figure S5B). Thus, the presence of 7-mer ARE motifs in the upregulated genes seems to better capture the preference of ZFP36L2 to target mRNA 3′UTRs with one or more 7-mer AREs.

Because of the importance of the 7-mer sequence for ZFP36L2 binding, we decided to visualize the distribution of 7-mers as a function of the |log_2_FC| >1 obtained from our RNA-seq analysis of the differentially expressed genes from the L2-fKO mouse model (Figure 3D). *Fgf23* mRNA was the most highly upregulated transcript (|log_2_FC| = 9.18) in the spleen, which we confirmed by qRT-PCR (600-fold increase, Supplementary Figure S4). However, the *Fgf23* transcript contains no 7-mer AREs and the only ARE (which is a 5-mer) does not bind to ZFP36L2 (Figure 3A). We therefore decided to further validate the *Elavl2* (*HuB* or *Mel-N1*) for the reasons outlined below.

### Modulation of *Elavl2* mRNA by ZFP36L2

**Figure 4.**
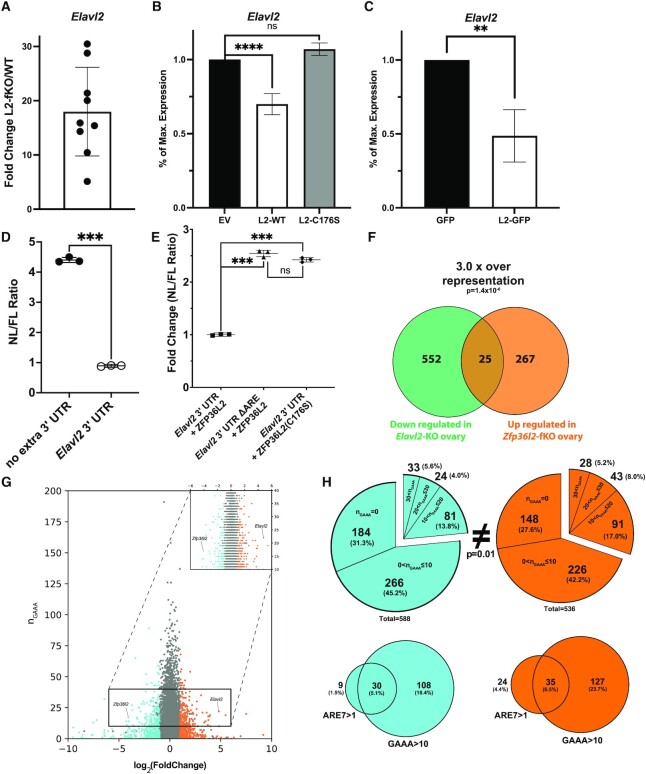
Modulation of *Elavl2* mRNA by ZFP36L2. (**A**) Fold Change of *Elavl2/Gapdh* mRNA expression in spleen from L2-fKO mice (*n* = 9) compared with the average expression in wild-type mice (*n* = 7). (**B**) Overexpression of ZFP36L2 (L2-WT, *n* = 5) decreased the expression of endogenous *Elavl2* mRNA in U2-OS cells compared to an empty vector (EV, *n* = 3), whereas the RNA-binding mutant of ZFP36L2 (L2-C176S, *n* = 3) did not, as measured by qRT-PCR. (**C**) Overexpression of ZFP36L2-GFP fusion (L2-GFP, *n* = 5) significantly downregulated endogenous *Elavl2* mRNA in U2-OS cells compared with overexpressed GFP (GFP, *n* = 3). (**D**) HEK 293 cells were co-transfected with firefly (FL, pGL4.5) and nanoluciferase (NL, pNL3.2.CMV) vectors; protein extracts were collected for dual luciferase measurements from three biological replicates (shown as circles). NL/FL ratios were computed from the two conditions, one where the nanoluciferase vector contained no additional 3′UTR (left) and another where the nanoluciferase vector contained the full-length of *Elavl2* 3′UTR (right). (**E**) HEK 293 cells were simultaneously transfected with three vectors: firefly (FL, pGL4.5) + nanoluciferase (NL, pNL3.2.CMV) + ZFP36L2 (pCMV.BGH3′/BS+). Protein extracts were collected for dual luciferase measurements from three biological replicates. When the 7-mer ARE binding sites were deleted (*Elavl2* 3′UTR ΔARE) it resulted in a 2.5-fold increase of the NL/FL ratio in comparison to the *Elavl2* 3′UTR in the presence of ZFP36L2 (triangles versus squares). A similar effect was observed when the mutant version of ZFP36L2-C176S was transfected with the NL reporter containing the full-length of *Elavl2* 3′UTR. Values for A–E are mean ± SD and *P* values were calculated using Student's *t*-test, ****P < 0.0001, ***P < 0.001 and **P < 0.01. (**F**) The green circle represents transcripts previously found to be down regulated in the ELAVL2-KO ovary (46) and the orange circle represents those upregulated in the L2-fKO ovary. Twenty five genes are common to both sets, this number of overlapping genes is significantly different than what would be expected by chance using (*P* < 1.4 × 10^–5^). (**G**) Number of GAAA (*n*_GAAA_) motifs in the longest 3′UTR of down- (cyan), unchanged (gray), and upregulated (orange) differentially expressed genes in the spleen of L2-fKO mice as a function of their log_2_ Fold Change. Inset (top-right corner) is magnification of 10 < n_GAAA_< 40. (**H**) Analyses of the number of GAAA (n_GAAA_) and 7-mer ARE motifs in the down- and upregulated genes in the spleen. Pie charts indicate the motifs in different subgroups: *n*_GAAA_= 0, 0 < *n*_GAAA_$ \le$10, 10 < *n*_GAAA_$ \le$20, 20 < *n*_GAAA_$ \le$30 and *n*_GAAA_ > 30 motifs in 3′ UTRs of down- (cyan) and upregulated (orange) genes. The distributions were compared using Kolmogorov–Smirnov test and were significantly different (*P* = 0.01), particularly a higher fraction of genes had *n*_GAAA_> 10 in the upregulated genes compared to the downregulated genes (30.2% versus 24.4%, respectively). Further analysis (Venn diagrams) of genes containing two or more 7-mer AREs and 11 or more GAAA motifs in their 3′UTRs did not reveal a difference in the co-occurrence of these motifs between the up- and downregulated lists.

The mRNA for *Elavl2* was significantly upregulated in the L2-fKO spleen (|log_2_FC| = 4.8, *P*_adj_ = 2.3e^–16^). Quantitative PCR confirmed the RNA-seq results and revealed an 18-fold increase in *Elavl2* mRNA in the L2-fKO spleen (Figure 4A). *Elavl2* mRNA contains three 7-mer AREs, and ZFP36L2 bound to all three 7-mer AREs in gel-shift assays (Figure 3A). ELAVL2 is an RNA-binding protein that binds GAAA sequence motifs in 3′UTRs (42,43), which stabilizes mRNA and/or enhances their translation, functions that are opposite to that of ZFP36L2.

To test whether *Elavl2* is a direct ZFP36L2 target, we overexpressed ZFP36L2 in U2-OS cells which is one of the 17 cell lines known to endogenously express *Elavl2* (31) and measured the *Elavl2* levels by qRT-PCR. ZFP36L2 overexpression led to a 30% decrease in *Elavl2* mRNA levels (*P* < 0.0001; Figure 4B), whereas a mutant version of ZFP36L2 that lacked RNA-binding properties (L2-C176S) did not alter *Elavl2* mRNA expression levels. Similarly, overexpression of ZFP36L2 fused with GFP resulted in a 50% decrease in *Elavl2* mRNA levels (*P* = 0.0028) compared with overexpression of GFP (Figure 4C). However, transfection efficiency of U2-OS cells can be low. We tested the transfection efficiency of U2-OS cells with a GFP construct and observed ∼50–60% of U2-OS cells were transfected under our conditions (Supplementary Figure S10). Thus, overexpression of ZFP36L2 using two different constructs led to decreased levels of endogenous *Elavl2* mRNA, suggesting that *Elavl2* is a direct target of ZFP36L2.

We next developed a reporter gene assay to test if ZFP36L2 downregulation of *Elavl2* is dependent on the 3′UTR of this transcript. For that we used a luciferase assay in which the 3′UTR of *Elavl2* (∼2500 bp) was added immediately downstream of the coding sequence of a nanoluciferase vector. When HEK 293 cells were transfected with the NL-*Elavl2* 3′UTR, a consistent and significant 4.5× fold decrease of nanoluciferase/firefly luciferase ratio was observed relative to the nanoluciferase construct with its own 3′UTR (Figure 4D). To further test if this effect is dependent on the presence of the 7-mer AREs, we created another nanoluciferase construct in which all three 7-mer ARE binding sites were deleted (*Elavl2* 3′UTR ΔARE). The NL-*Elavl2* 3′UTR ΔARE construct resulted in a 2.5x fold increase of the nanoluciferase/firefly luciferase (NL/FL) ratio in comparison to the *Elavl2* 3′UTR in the presence of ZFP36L2 (Figure 4E). As expected, when the mutant version of ZFP36L2-C176S was co-transfected with a nanoluciferase vector containing the *Elavl2* 3′UTR, the NL/FL ratio increased to similar levels as when the AREs were removed (Figure 4E). These assays confirm that the effect of ZFP36L2 on *Elavl2* mRNA is dependent on the presence of AREs of the 7-mer type and on the functional zinc finger domain of ZFP36L2.

The discovery that *Elavl2* mRNA is a target of ZFP36L2 suggests that these two RBPs may act in concert to regulate genes. ELAVL2 is known to be an ARE-RBP that preferentially binds to transcripts containing GAAA motifs in their 3′UTR (42), leading to stabilization and promotion of translation of their target transcripts (6,44,45). ELAVL2 stabilizes its own transcript through direct binding to GAAA motifs present in its own 3′UTR (42). To investigate if these two RBPs act in concert in the same set of genes, we performed statistical overlap analysis of previously published mRNAs found to be downregulated in the ELAVL2-KO ovary (46) and our list of upregulated genes in the L2-fKO ovary (Figure 4F). Among these two data sets, we identified 25 genes. To determine if this overlap is higher than what is expected by chance we used a hypergeometric distribution normal approximation to compute the probability of overlap as described in (47) and determined a 3.0-fold increase of the overlap representation (*P* = 1.4 × 10^–6^). This statistically significantly higher than expected overlap suggests that ZFP36L2 and ELAVL2 act on some common mRNA targets. We also visualized the frequency distribution of GAAA motifs in 3′UTRs of the up-, down-, and unchanged genes in our L2-fKO RNA-seq data (Figure 4G). The frequency of occurrence of the GAAA motif is high in the mouse genome, with over 80% of 3′UTRs containing at least one GAAA motif. As such, we opted to subgroup GAAA motifs into five categories from *n*_GAAA_ = 0 to *n*_GAAA_ > 30, where *n*_GAAA_ was the number of motifs in each 3′UTR (Figure 4H). When we compared the distributions of the GAAA motifs in the down- (cyan) and upregulated (orange) genes from the L2-fKO spleen RNA-seq data, we observed small, but significant (*P* = 0.01), differences in the distribution of GAAA motifs in the upregulated genes compared with the downregulated genes (Figure 4H, upper pie charts). The most obvious difference in the distributions was the increased proportion of transcripts compiled in the upregulated genes whose 3′UTRs contained more than 10 GAAA motifs (30.2% versus 23.4%, up- and downregulated respectively).

It is not known whether ELAVL2 preferentially targets multiple GAAA repeats. However, a hallmark of the specificity of many RNA-binding proteins is a preference for target RNAs with motif repeats. When we consider the overlap of genes with 3′UTRs that contained more than one 7-mer UAUUUAU and more than 10 GAAA motifs (Figure 4H, lower Venn diagrams), we do not observe significant differences in the overlap for up- and downregulated genes, likely because few genes simultaneously contain the ARE 7-mer and more than 10 GAAA motifs. We may simply not have the statistical power to resolve these effects in our current data set. Importantly, *Elavl2* was not identified as differentially expressed in the two previous transcriptome studies in erythrocyte lineages (25) and oocytes (24), which suggests that ZFP36L2 targeting of *Elavl2* mRNA is specific to the spleen.

## DISCUSSION

Like various RNA-binding proteins, ZFP36L2 is ubiquitously expressed in many tissues. Mouse models with disruptions of the *Zfp36l2* gene have been created to assess the function(s) of this protein. Two models revealed a female infertility phenotype (20,24); however, biologically significant effects of *Zfp36l2* knockdown were observed in other tissues, including red blood cell differentiation (23,25). This suggests that the physiological role of ZFP36L2 is likely broader than just its role in female infertility. Here we performed transcriptome profiling to examine the function of ZFP36L2 in the spleen from mouse pups lacking this protein during the first ten days after birth. Two other transcriptome analyses have been reported: a microarray analysis of an *ex vivo* mouse model of erythrocyte differentiation, wherein *Zfp36l2* was knocked down (25), and a single-cell RNA-seq analysis of oocytes lacking *Zfp36l2* (24).

While 75% of genes that we detected in the spleen by RNA-seq were also detected in oocytes by scRNA-seq, only 24 of the upregulated genes were common in both tissues, which corresponded to 5% of all upregulated genes in the spleen. A similar pattern was observed when we compared spleen and the erythrocyte lineage, which shared 50% of the same expressed genes but only nine genes were upregulated in both tissues, about 2% overlap.

Therefore, the genes that were differentially expressed in the spleen data set were unique from those expressed in erythrocyte lineage and oocytes. Even using the same biological model, only minimal overlap in the upregulated genes was observed in the spleen and ovary of the L2-fKO mouse (Supplementary Figure S5A). This result is surprising since statistically we would expect a similar level of overlap in the differentially regulated and unchanged genes. Intriguingly, these overlap percentages are below the 8% estimated proportion of mRNAs that would contain functional AREs in the whole human genome using ARED (10). These data suggest that other tissue factors modulate ZFP36L2-mRNA targeting. Importantly, the mRNAs modulated by ZFP36L2 in oocytes (24) that affect histone methylation are not differentially regulated in the spleen, which suggests that ZFP36L2-targeting is different across tissues.

Nevertheless, in all tissue studied thus far, spleen, ovary, erythrocyte lineage and oocytes, the upregulated genes were enriched in AREs, particularly when evaluated by the ARE*Score* program. This enrichment suggested that 3′UTR AREs are a hallmark of ZFP36L2 binding in all four tissues. However, the metric computed by the ARE*Score* algorithm was optimized based on targets of ZFP36 (30), which preferentially binds to overlapping 5-mers or blocks of overlapping 5-mers such as those found in the 3′UTR of *Tnf-α* or *Gm-csf* mRNAs (33). To investigate mRNA targets specific to ZFP36L2, we performed gel shift assays with 12 new RNA probes that contained only one ARE motif and combined these results with published gel shift assays of an additional 15 probes. We used gel shift assays to directly validate specific binding, instead of crosslinking or immunoprecipitation assays for which it is more difficult to exclude nonspecific interactions. In addition, as a control for nonspecific interactions, we used the C176S mutant in the zinc finger domain of ZFP36L2 that is unable to bind the ARE (12). Our gel shift results showed that ZFP36L2-binding required a minimum of a 7-mer (UAUUUAU), and a probe containing a single 5-mer (AUUUA) seems to be insufficient (Figure 3A). Indeed, when we measured the 7-mer enrichment, we did observe a statistically significant enrichment of this motif only in the upregulated genes in the L2-fKO spleen (Figure 3C). Although, in gel-shift shift assays ZFP36 did not bind to probes containing a single 7-mer ARE (Supplementary Figure S9), we cannot unequivocally exclude binding based on this assay alone, as its binding affinity maybe below the limit of detection of this technique. Thus, this difference is only suggestive that these two proteins have different binding preferences, and more studies are necessary to further investigate the possibility of lower affinity binding. Our results suggest a subtle but important difference in the targeting specificity of ZFP36L2 compared with ZFP36, and we propose that computing the density of 7-mer AREs in the 3′UTR might be a valuable approach to identifying ZFP36L2 targets. Therefore, we reexamined our list of upregulated genes for those containing multiple 7-mer AREs and identified a particularly interesting RNA-binding protein, *Elavl2* (*HuB* or *Mel-N1*), as a putative novel target of ZFP36L2.

Several lines of evidence support ZFP36L2 regulation of *Elavl2* mRNA. Overexpression of ZFP36L2 significantly downregulates *Elavl2* mRNA, whereas overexpression of the non-binding C175S mutant of ZFP36L2 does not. Overexpression of ZFP36L2 reduces the expression of *Elavl2* by 30–50%. This partial effect, is likely due to other limiting factors also required for this degradation process (48). Additionally, our nanoluciferase/firefly reporter assay experiments demonstrated that the ZFP36L2 effect on *Elavl2* is dependent on the 3′UTR sequence of *Elavl2* and requires the 7-mer AREs and the integrity of the tandem zinc finger domain of ZFP36L2 to result in *Elavl2* decay. The discovery that *Elavl2* mRNA is a target of ZFP36L2 suggests that these two RBPs may act in concert to regulate genes. In fact, the number of potential ELAVL2 mRNA targets were significantly increased in our upregulated genes (Figure 4F). Interestingly, we observed an increased fraction of transcripts compiled in the upregulated genes in which the 3′UTRs contained >10 GAAA motifs. On the other hand, *Zfp36l2* mRNA 3′UTR contains multiple GAAA motifs (Figure 4G), which are known target motifs of ELAVL2, suggesting a feedback regulatory mechanism between the two RNA binding proteins by modulation through their respective mRNAs. Indeed, previous immunoprecipitation of ELAVL2 protein reported *Zfp36l2* mRNA as a potential ELAVL2-target (46). Structural analysis of the 3′UTR mRNAs which simultaneously contain multiple 7-mer AREs and 10 GAAA motifs would be helpful to further understand the molecular basis of co-regulation by ZFP36L2 and ELAVL2.

The ELAV-like RNA-binding protein family has four members in vertebrates, ELAVL1-4. ELAVL2, ELAVL3 and ELAVL4 are cytosolic and expressed mainly in neurons, whereas ELAVL1 is expressed ubiquitously and present predominantly in the nucleus. These proteins share high homology between their three RNA recognition motifs (RRMs), but they differ significantly in their amino-terminal ends and at the so-called ‘hinge domain’, a region between the second and third RRMs. These proteins are involved in post-transcriptional control of their mRNA targets, usually leading to mRNA stabilization and enhanced translation (6,44,45), effects opposite to ZFP36L2, which also happens to be a cytosolic protein (21,49). Interestingly, other investigators have reported opposite effects of ELAV and ZFP36-like family members on mRNA transcripts. For example, ELAVL1 and ZFP36 have antagonistic effects on the stability of IL-3 (50) and IL-8 mRNAs (51). Thus, an opposing effect between ELAVL2 and ZFP36L2 is analogous to this observation. However, the antagonistic action between these two proteins presented here has not been previously investigated.

Even though, we observed an 18-fold increase in *Elavl2* mRNA in L2-fKO, *Elavl2* expression in the wild-type spleen is remarkably low (3.0 ± 2.7 TPM), below the detectable levels of Northern or immunoblot assays in splenic samples (not shown). This low expression explains the limited stabilization potential of ELAVL2, under normal conditions, in the spleen. The *Elavl2* mRNA expression in the L2-fKO spleen was ∼54 TPM, which is still considered below moderate levels. ELAVL2 expression in the wild-type spleen is probably maintained at a low level by ARE-mediated ZFP36L2 induced degradation. Most ZFP36L2 target mRNAs are expressed at high levels (25); however, *Elavl2* does not belong to this category because of its low expression in the spleen. In the brain, where ELAVL2 is highly expressed, ZFP36L2 is barely detectable in normal conditions (21); thus, it is not surprising that there are no major effects on *Elavl2* expression in the total brain in the absence of ZFP36L2 (Supplementary Figure S11).

Interpreting the observed differences in the distributions of RNA motifs is complex because the cell compositions of the L2-fKO spleens seem to be different than the wild type (i.e. there is a decrease number of erythrocyte lineages (Figure 1D) and because of the compounding ELAVL2 stabilization effect on some ARE containing transcripts. Future single cell RNA-seq analyses on specific cell populations combined with deconvolution of bulk RNA-seq data (52) will yield further insight into these intricate networks of post-transcriptional regulation.

Our investigation of the tissue specificity of ZFP36L2 targeting reveals a complex network of post-transcriptional regulation. ZFP36L2 seems to have a different ARE-targeting profile compared with the prototypical family member, ZFP36. It is likely that each ZFP36 family member has subtle differences in their binding specificity, which ultimately dictates their function. The lack of overlap between upregulated genes in the four transcriptome profiling experiments performed to date, two from this work and (24,25), indicates a high level of tissue specificity in ZFP36L2 targeting *in vivo*. Although, meta-analysis of differential gene expression can identify common features of RNA-binding protein targets, it is difficult to use these general features to predict functional targets. Careful biochemical confirmation of binding combined with effects of overexpression are necessary to establish novel specific targets of RNA-binding proteins. A particularly interesting aspect ZFP36L2-targeting of *Elavl2* mRNA is that these two genes are not co-expressed post-development in the same tissues. In the spleen, it appears that *Elavl2* is effectively suppressed by ZFP36L2, and this suppression may function to prevent ELAVL2 from stabilizing some specific RNAs. It is therefore clear that tissue specificity of a given RNA binding protein is driven in part by the presence or absence of other RNA binding proteins in these tissues, thus revealing these complex interaction networks is key to understanding the physiological roles of this central class of proteins.

## ACCESSION NUMBERS

Differential expression results and RNA-seq reads are provided in the gene expression omnibus (GEO Accession ID GSE168729).

## Supplementary Material

gkac209_Supplemental_File
