# Supplementary material for: Sequence and tissue targeting specificity of ZFP36L2 reveals Elavl2 as a novel target with co-regulation potential
Source: Nucleic Acids Res. 2022 Apr 5;50(7):4068–82. doi: 10.1093/nar/gkac209 (PMC9023260; doi:10.1093/nar/gkac209)
Supplement: gkac209_Supplemental_File [file gkac209_supplemental_file.pdf]

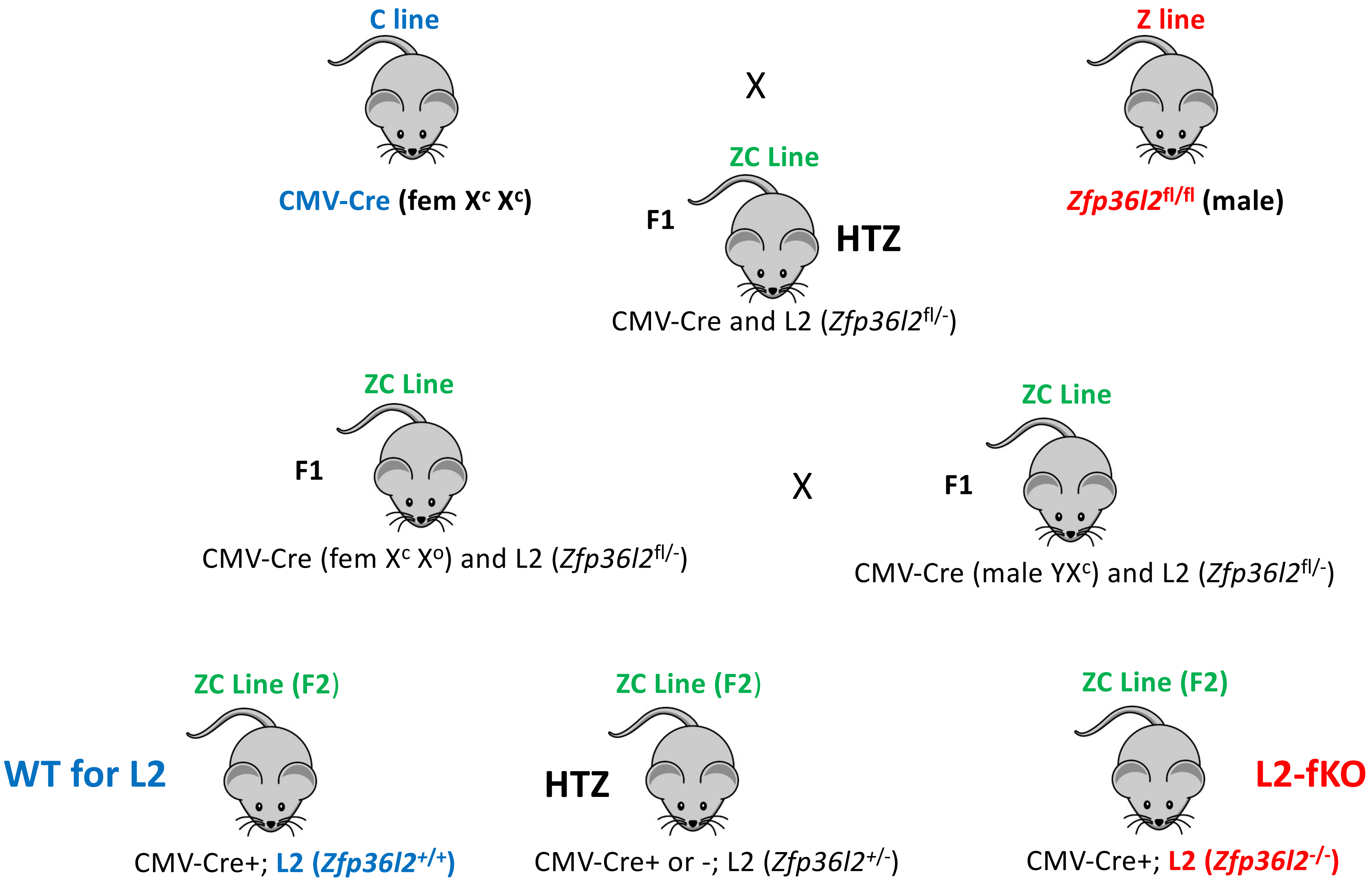

C line
CMV-Cre (fem X^c X^c)
X
Z line
Zfp36l2^fl/fl (male)
ZC Line
F1
HTZ
CMV-Cre and L2 (Zfp36l2^fl/-)
ZC Line
F1
CMV-Cre (fem X^c X^o) and L2 (Zfp36l2^fl/-)
X
ZC Line
F1
CMV-Cre (male YX^c) and L2 (Zfp36l2^fl/-)
ZC Line (F2)
WT for L2
CMV-Cre+; L2 (Zfp36l2^+/+)
ZC Line (F2)
HTZ
CMV-Cre+ or -; L2 (Zfp36l2^+/-)
ZC Line (F2)
L2-fKO
CMV-Cre+; L2 (Zfp36l2^-/-)

Figure S1

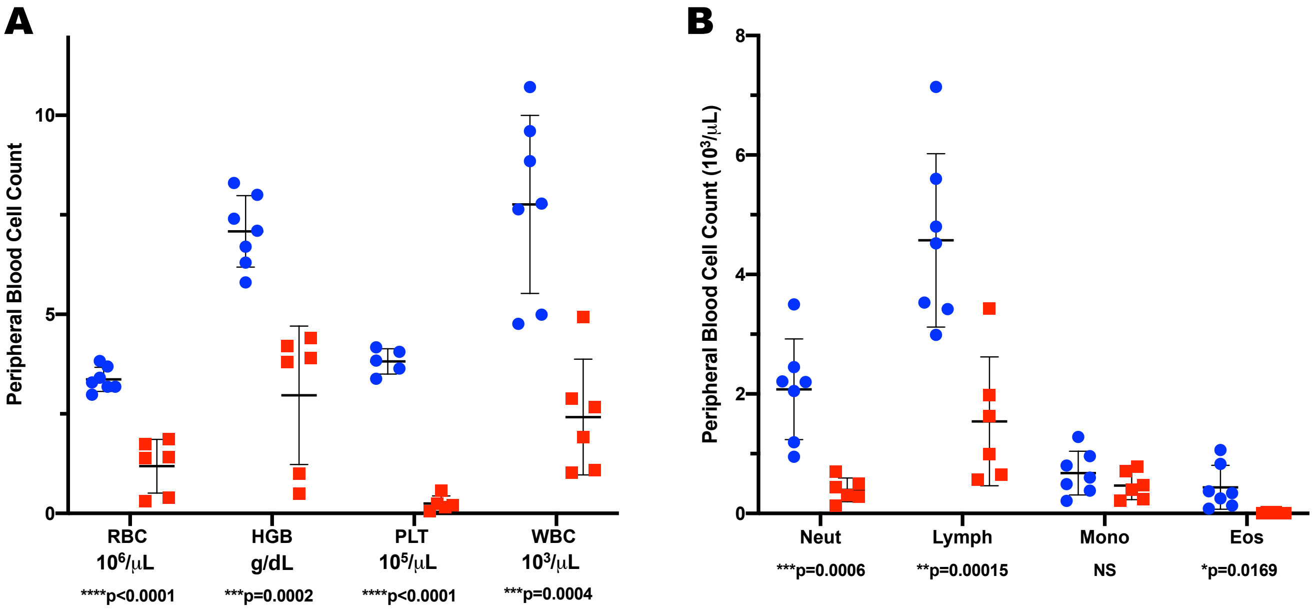
A
B
Peripheral Blood Cell Count
10
5
0
RBC
10⁶/μL
****p<0.0001
HGB
g/dL
***p=0.0002
PLT
10⁵/μL
****p<0.0001
WBC
10³/μL
***p=0.0004
Peripheral Blood Cell Count (10³/μL)
8
6
4
2
0
Neut
***p=0.0006
Lymph
**p=0.00015
Mono
NS
Eos
*p=0.0169

Figure S2

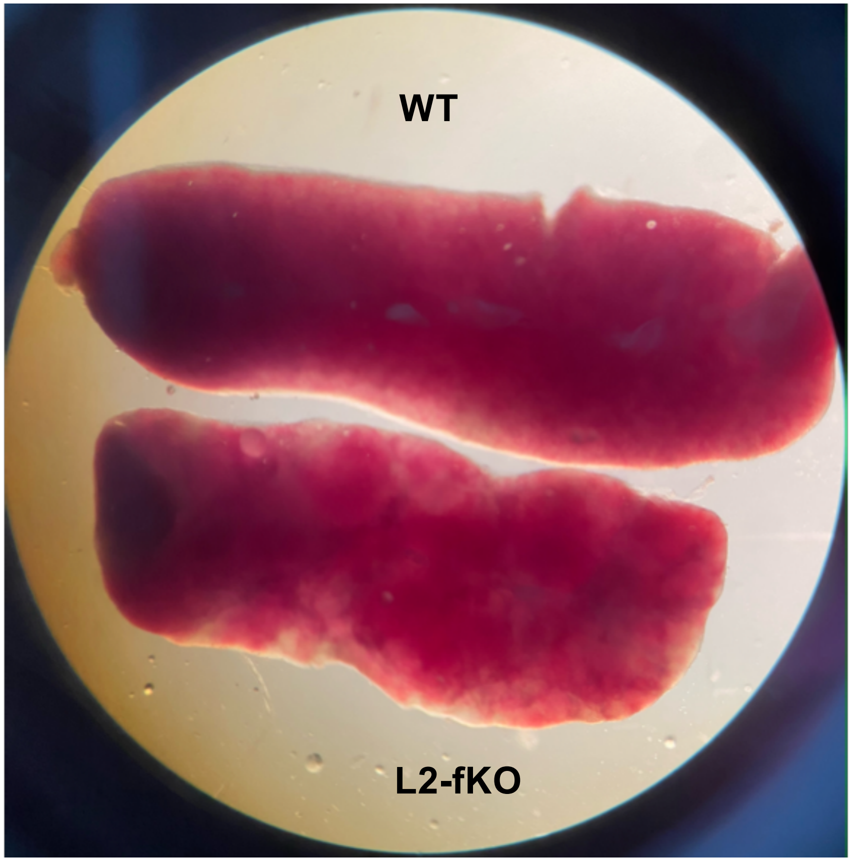

Figure S3

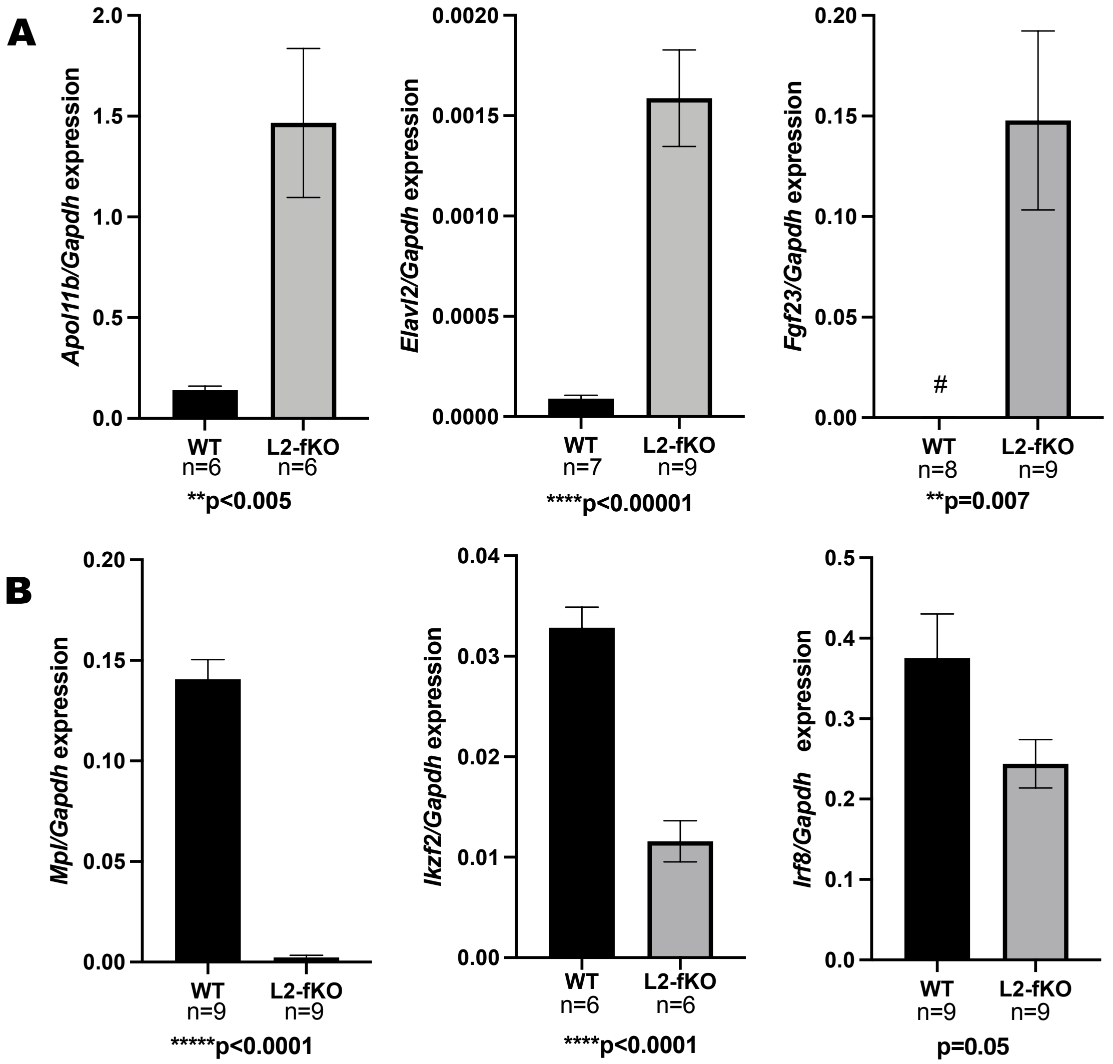

A
Apol11b/Gapdh expression
2.0
1.5
1.0
0.5
0.0
WT
n=6
L2-fKO
n=6
**p<0.005
Elavl2/Gapdh expression
0.0020
0.0015
0.0010
0.0005
0.0000
WT
n=7
L2-fKO
n=9
****p<0.00001
Fgf23/Gapdh expression
0.20
0.15
0.10
0.05
0.00
#
WT
n=8
L2-fKO
n=9
**p=0.007
B
Mpl/Gapdh expression
0.20
0.15
0.10
0.05
0.00
WT
n=9
L2-fKO
n=9
*****p<0.0001
Ikzf2/Gapdh expression
0.04
0.03
0.02
0.01
0.00
WT
n=6
L2-fKO
n=6
****p<0.0001
Irf8/Gapdh expression
0.5
0.4
0.3
0.2
0.1
0.0
WT
n=9
L2-fKO
n=9
p=0.05

Figure S4

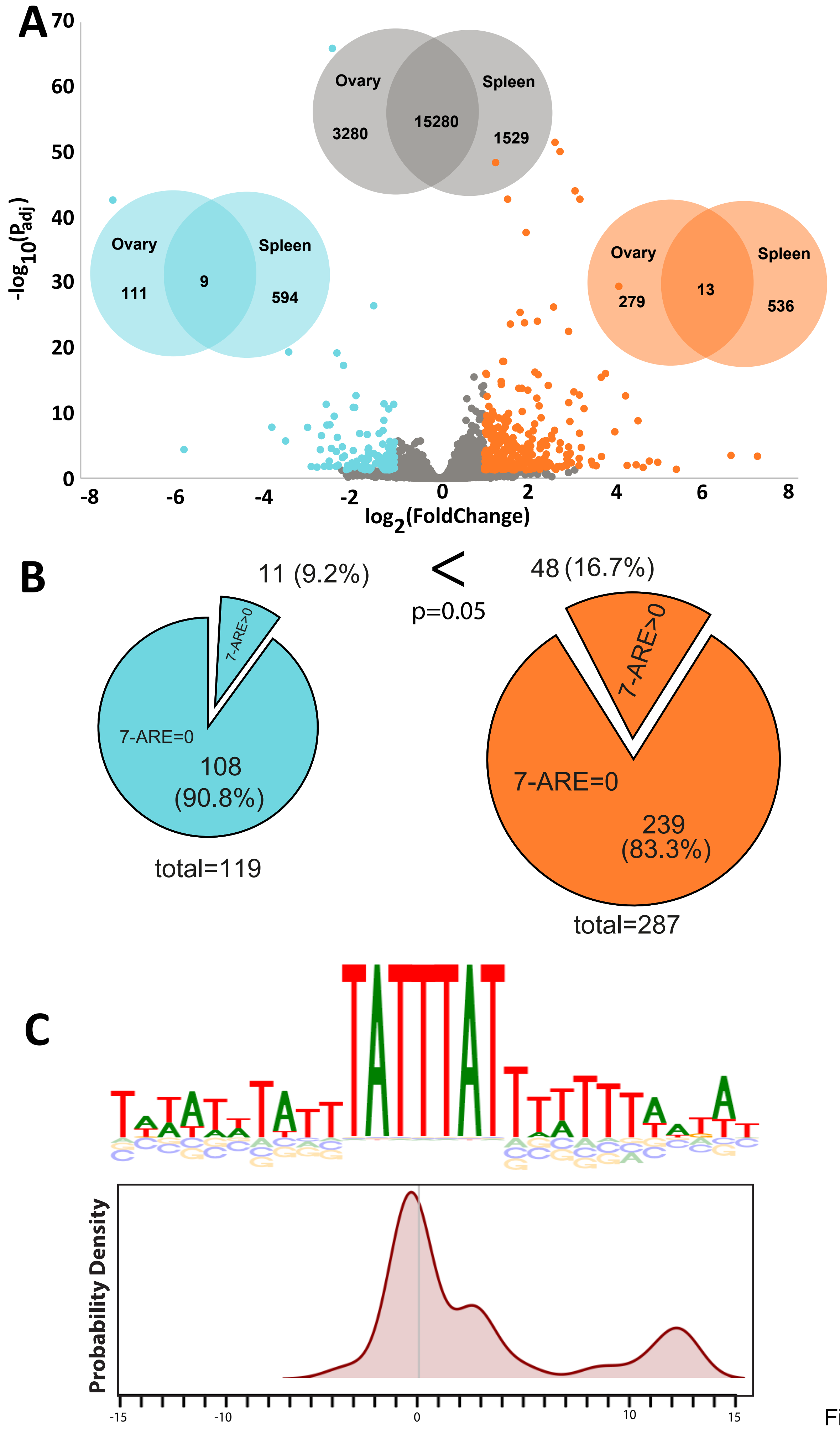
A
70
60
50
40
30
20
10
0
-8
-6
-4
-2
0
2
4
6
8
log2(FoldChange)
-log10(Padj)
Ovary
Spleen
3280
15280
1529
Ovary
Spleen
111
9
594
Ovary
Spleen
279
13
536
B
11 (9.2%)
<
p=0.05
48 (16.7%)
7-ARE>0
7-ARE=0
108
(90.8%)
total=119
7-ARE>0
7-ARE=0
239
(83.3%)
total=287
C
Probability Density
-15
-10
0
10
15

Figure S5

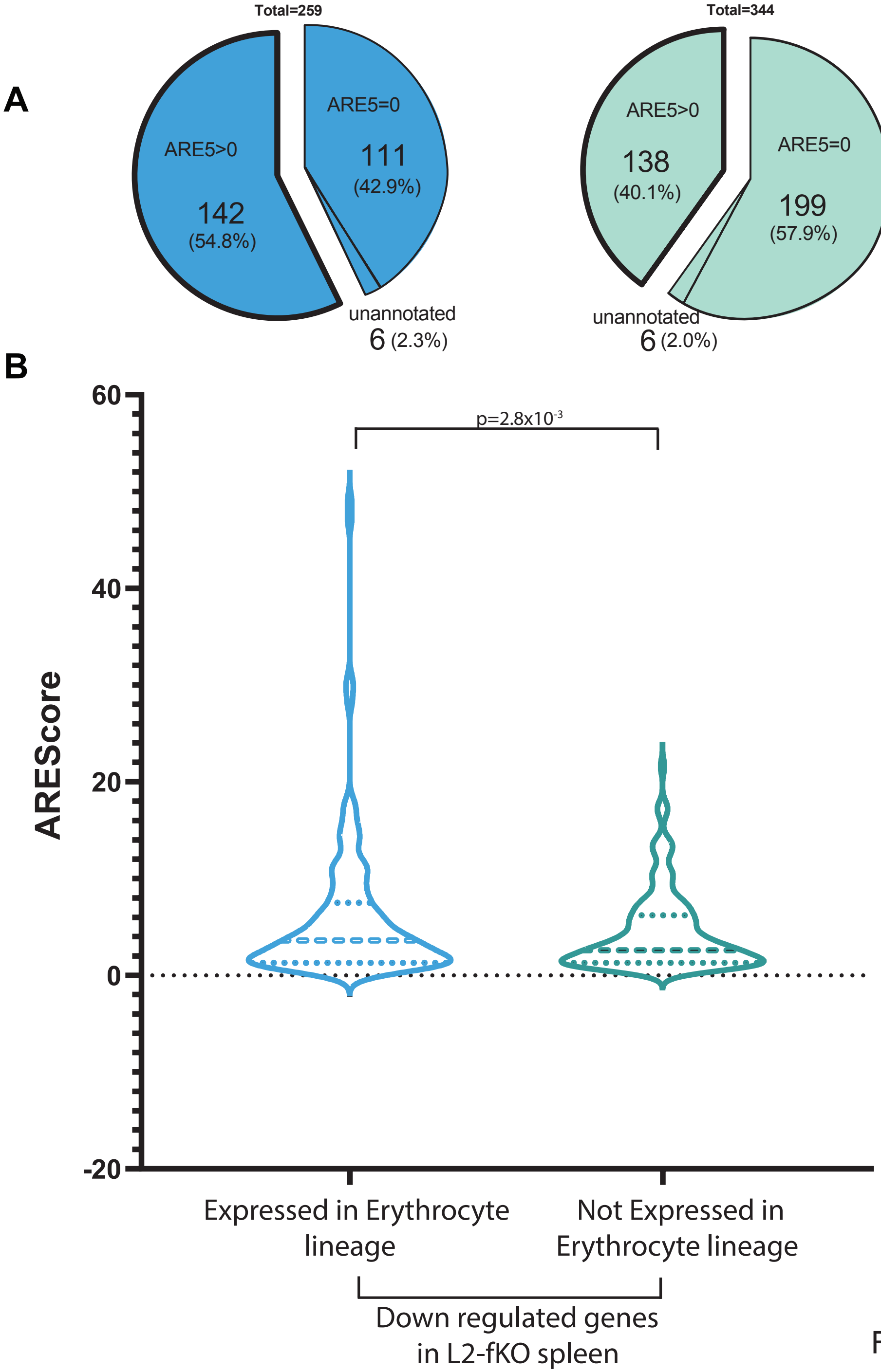
A
Total=259
ARE5>0
142
(54.8%)
ARE5=0
111
(42.9%)
unannotated
6 (2.3%)
Total=344
ARE5>0
138
(40.1%)
ARE5=0
199
(57.9%)
unannotated
6 (2.0%)
B
AREScore
60
40
20
0
-20
p=2.8x10-3
Expressed in Erythrocyte lineage
Not Expressed in Erythrocyte lineage
Down regulated genes in L2-fKO spleen

Figure S6

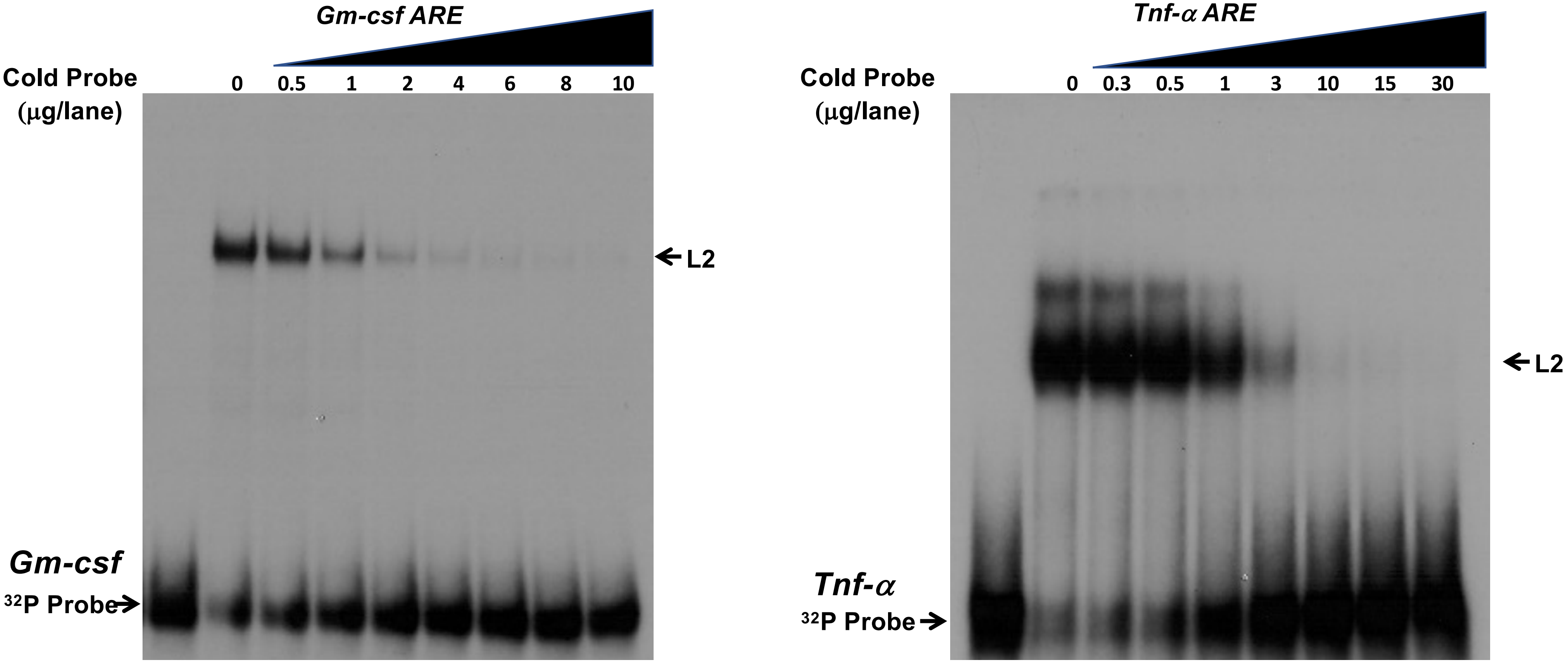

Gm-csf ARE
Cold Probe (μg/lane)
0 0.5 1 2 4 6 8 10
L2
Gm-csf
32P Probe
Tnf-α ARE
Cold Probe (μg/lane)
0 0.3 0.5 1 3 10 15 30
L2
Tnf-α
32P Probe

Figure S7

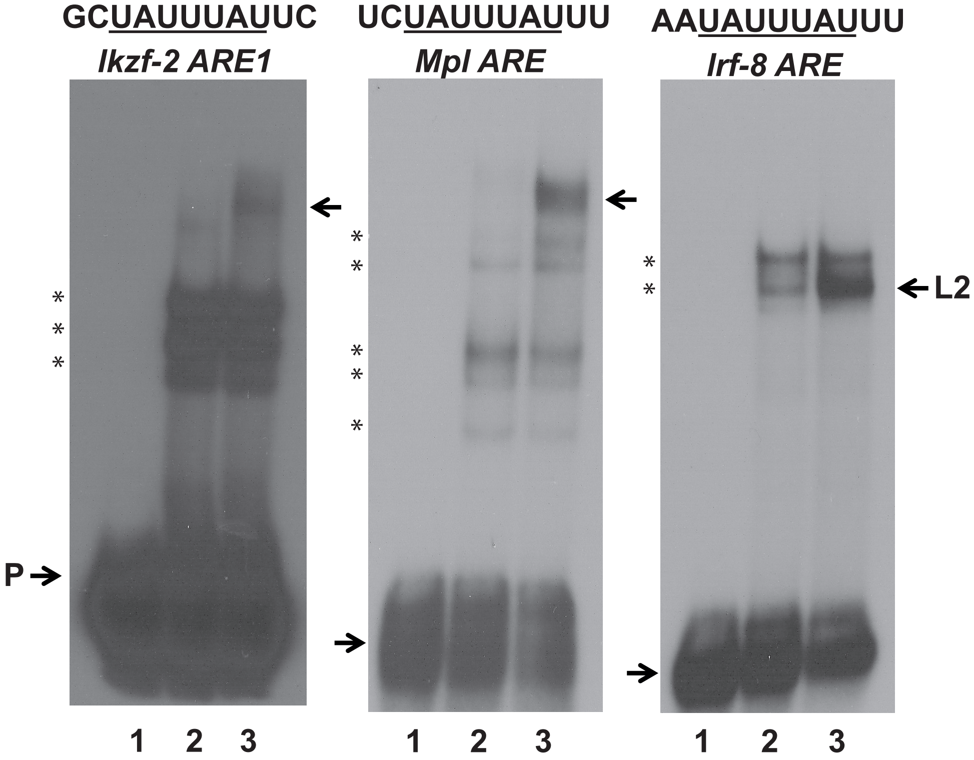

GCUAUUUAUUC
Ikzf-2 ARE1
UCUAUUUAUUU
Mpl ARE
AAUAUUUAUUU
Irf-8 ARE
P
L2
1 2 3
1 2 3
1 2 3

Figure S8

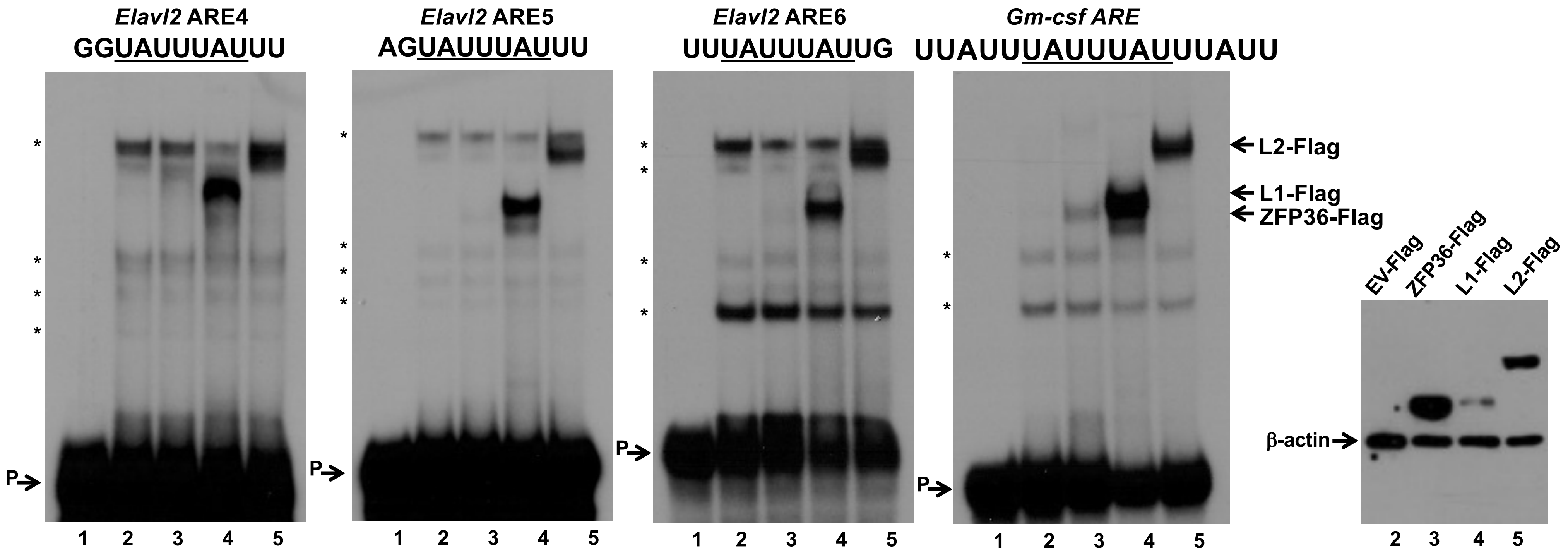

Elavl2 ARE4
GGUAUUUAUUU
Elavl2 ARE5
AGUAUUUAUUU
Elavl2 ARE6
UUUAUUUAUUG
Gm-csf ARE
UUAUUUAUUUAUUUAUU
L2-Flag
L1-Flag
ZFP36-Flag
EV-Flag
ZFP36-Flag
L1-Flag
L2-Flag
β-actin
P
1 2 3 4 5
2 3 4 5

Figure S9

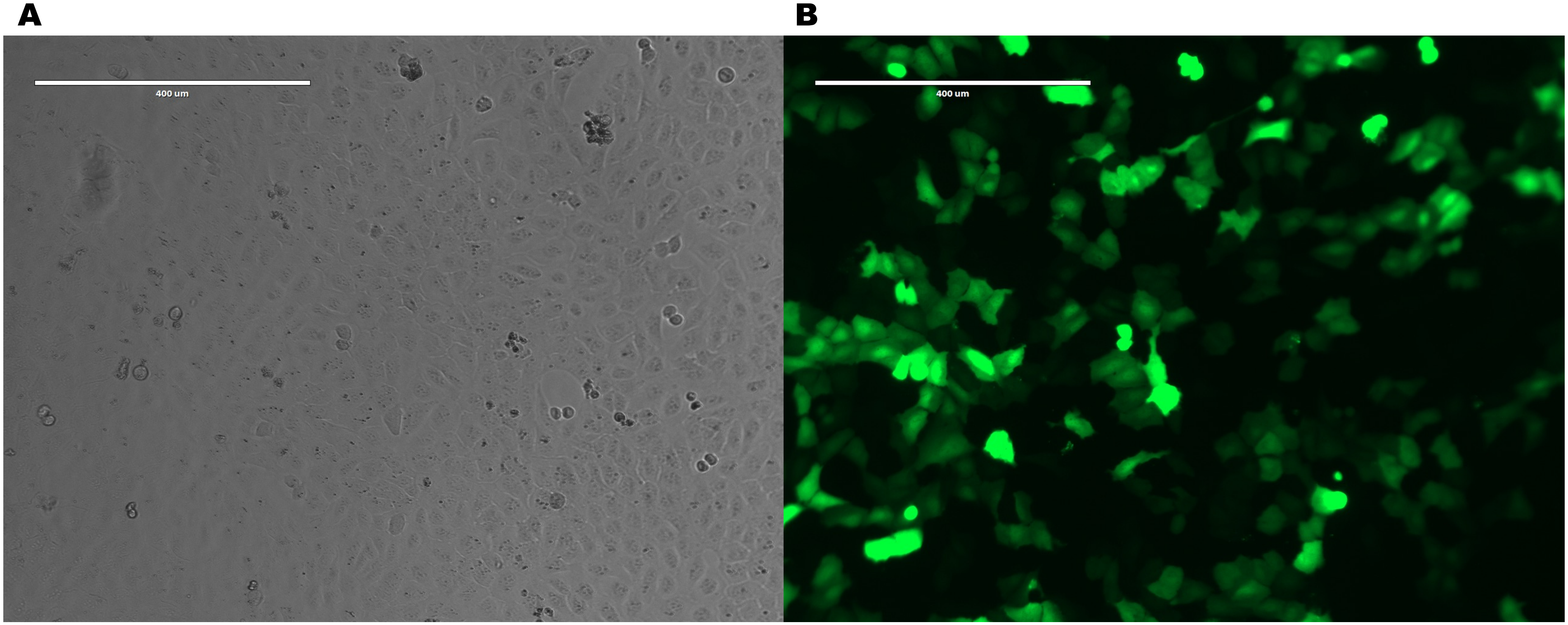

Figure S10

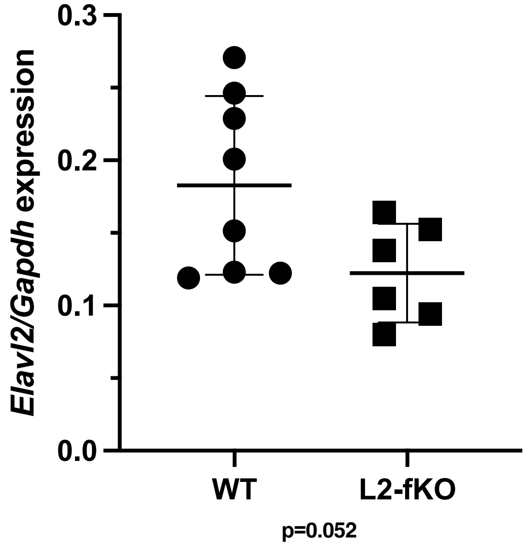
0.3
0.2
0.1
0.0
Elavl2/Gapdh expression
WT
L2-fKO
p=0.052

SUPPLEMENTARY DATA

Supplemental Figure 1: Schematic crossing approach to generate the conditional mouse model lacking *Zfp36l2* in all tissues (L2-fKO). A conditional knockout of *Zfp36l2* was obtained by crossing CMV-Cre transgenic females (shown as line C) with the *Zfp36l2* floxed males (*Zfp36l2*$^{fl/fl}$, represented as Line Z) to generate *Zfp36l2*$^{fl/-}$; CMV-Cre in the first generation (F1). When *Zfp36l2*$^{fl/-}$; CMV-Cre (F1) females were mated with *Zfp36l2*$^{fl/-}$; CMV-Cre (F1) males, animals of both sex with both *Zfp36l2* recombined alleles were generated in the second generation (*Zfp36l2*$^{-/-}$; CMV-Cre). These animals are referred as L2-fKO. Animals carrying both wild type copies of *Zfp36l2* and the Tg.CMV-Cre were used as wild type littermate controls for experiments.

Supplemental Figure 2: Analysis of circulating hematological cells during the first week of mouse life. A) Red blood cells (RBC), hemoglobin (HGB) and white blood cells (WBC) were counted from peripheral blood from wild type (blue circles, n=7) and L2-fKO (red squares, n=6) mice from neonatal day 6 to day 8 animals; counts for platelets (PLT) were derived from five animals in each group. **B)** White blood cell differential counts were obtained from these same animals. Horizontal prominent central bars are mean, vertical bars are ± SD, and *p* values were calculated using Student's t-test.

Supplemental Figure 3: Macroscopic anatomy of the spleen. Representative splenic images from age-matched wild type and L2-fKO mice photographed at 3X magnification**.**

Supplemental Figure 4: Measurement of mRNA levels by qRT-PCR of selected up- and downregulated genes identified by RNA-seq. A) *Apol11b, Elavl2 and Fgf23* were expressed at significantly higher levels in the L2-fKO compared with wild type spleen (upper panel). **B)** Other genes, such as *Ikzf2*, *Mpl* and *Irf8*, despite containing a 7-mer (UAUUUAU) in their 3'UTRs, were expressed at decreased levels in L2-fKO. Values are mean ± SEM, and *p* values were calculated using Student's t-test.

Supplemental Figure 5: Differential gene expression analysis of L2-fKO ovaries and comparison with spleen. A) Volcano plot of differential gene expression of wild type (n=3) vs L2-fKO (n=3) ovaries, with cut off |log_2FC>1| and adjusted p-value<0.05. The 132 downregulated genes are colored blue, and the 287 upregulated genes are colored orange. The remaining 19,837 genes were not differentially expressed genes and are plotted in gray. The gray Venn diagram illustrates all genes expressed in ovary and spleen detectable by RNA-seq. Venn diagrams in blue and orange represent genes differentially down- and upregulated, respectively, in both tissues. **B)** Occurrence of 7-mer (UAUUUAU) AREs in the 3'UTR of genes detected in the ovary. Left and right pie chart corresponds to downregulated (blue) and upregulated (orange) genes in ovarian samples, respectively. Note that in the pie charts, poorly annotated transcripts, for which we could not identify a 3'UTR were excluded. *p* values were calculated using a Kolmogorov-Smirnov test. **C)** Sequence logo and probability density of 'de novo' motif discovery using

BaMMotif on the sequences of the 3'UTRs from the upregulated genes in the ovary that contained one or more 7-mer AREs.

Supplemental Figure 6: ARE*Score* analysis of downregulated genes in the L2-fKO spleen and also present in the erythrocyte lineage. Overlap between downregulated genes in the L2-fKO spleen and also expressed in the erythrocyte lineage (24) was computed and resulted in two lists of down regulated genes, i.e., genes either expressed (left) or not expressed (right) in the erythrocyte lineage. **A)** Among them, 259 genes were expressed in both the erythrocyte lineage and found to be downregulated in the present study (left pie chart, cyan); thus, these genes were considered erythrocyte lineage-specific. The other 344 genes were not expressed in erythrocyte lineage but were downregulated in the L2-fKO spleen and considered splenic-specific genes (green, right pie chart). The pie charts reveal that the overlapping genes (cyan) had a higher portion (54.8%) of at least one 5-mer ARE motifs in their corresponding 3' UTRs compared with the splenic-specific genes (40.1%). Note that in both charts we identified and counted the ncRNAs, which comprised about 2%, and which do have identifiable 3'UTRs. **B)** Overlapping genes, simultaneously expressed in the erythrocyte lineage and downregulated in the L2-fKO spleen (cyan violin plot), had statistically different distributions of ARE*Score* when compared with the non-erythrocyte lineage genes downregulated in the L2-fKO spleen (green violin plot); a Kolmogorov–Smirnov analysis was performed (p=2.8x10^{-3}). The ARE*Score* numbers were higher in the overlapping genes (cyan violin plot). This observation partially explained why we observed higher ARE*Scores* in the L2-fKO splenic downregulated genes (Figure 2D) because the cells that would express these genes, i.e., the erythrocyte lineages, were absent in the L2-fKO spleen.

Supplemental Figure 7: Competitive dose response of ZFP36L2 binding to two classical ZFP36 targets. Both RNA electrophoretic mobility shift assays were performed by incubating 0.2 × 10^5 cpm of labeled probes in the presence of increasing amounts of cold probe (shown on top) and fixed amount of protein extracts (10 µg per lane) expressing ZFP36L2. The migration of each probe in the absence of any added protein is shown in the first lanes and corresponds to *Gm-csf* ARE and *Tnf-α* probes, left and right, respectively. Arrows to the right indicate complexes containing ZFP36L2 and labeled probes.

Supplemental Figure 8: ZFP36L2 binding to additional RNA containing ARE probes. RNA electrophoretic mobility shift assays were performed by incubating 0.2 × 10^5 cpm of different ARE 7-mer probes, *Ikzf2* ARE1(left), *Mpl* (middle) and *Irf8* (right panel), with protein extracts (10 µg per lane) from HEK 293 cells transfected with a vector that expressed an RNA-binding mutant of ZFP36L2-C176S (lanes 2) or with a vector that expressed wild type ZFP36L2 (lanes 3). Lanes 1 show the migration of the probe in the absence of any protein. Incubation of protein extracts containing the ZFP36L2 RNA-binding mutant (lanes 2) produced nonspecific bands (indicated by asterisks). These nonspecific bands likely correspond to complexes of endogenous proteins present in HEK 293. Arrows to the right indicate complexes containing ZFP36L2 and labeled probes that contained a single ARE of the 7-mer type.

Arrows to the left point to the original migration of the probes (P) without any protein present. Shown at the top of each panel is a partial nucleotides sequence present in the ~30 nucleotide long probe, including the 7-mer ARE (underlined) flanked by adjacent nucleotides on each side.

Supplemental Figure 9: Binding of ZFP36 family members to RNA probes containing the 7-mer type ARE. RNA electrophoretic mobility shift assays were performed by incubating with 0.2 × 10^5 cpm of different ARE probes; *Elavl2* ARE4, *Elavl2* ARE5, *Elavl2* ARE6 and *Gm-csf,* with 10 µg of protein extract from HEK 293 cells transfected with a vector that express Flag, ZFP36-Flag, ZFP36L1-Flag or ZFP36L2-Flag (lanes 2, 3, 4 and 5, respectively). Shown at the top of each panel is a partial nucleotides sequence present in the ~30 nucleotide long probe, including the 7-mer ARE (underlined). Lane 1 of each gel shows the migration of the probe in the absence of any protein (P). Protein extracts derived the Flag empty vector (lanes 2) were incubated with each probe producing nonspecific bands (indicated by asterisks), which were also present in the other protein extracts. These nonspecific bands likely correspond to complexes of endogenous proteins present in HEK 293. Arrows indicate complexes containing ZFP36L1-Flag (L1) and ZFP36L2-Flag (L2) with labeled probes containing a single ARE of the 7-mer type. Note that ZFP36-Flag did not bind to any of the probes containing an isolated 7-mer ARE, but did bind to the *Gm-csf* probe which contains adjacent AREs. All protein extracts contained similar levels of proteins, except for higher expression of ZFP36-Flag as shown by immunoblotting (insert to the right).

Supplemental Figure 10: Transfection efficiency of U2-OS cells using a GFP construct. A) Bright field picture of U2-OS cells using a 10x magnification lens was taken 24 hours after transfection. **B)** The same field was then microphotography using 10x magnification fluorescent lens to visualize the of GFP transfected cells.

Supplemental Figure 11: Measurement of *Elavl2* mRNA levels in wild-type and L2-fKO brain by qRT-PCR. Total RNA was isolated from wild-type (circles, n=8) and L2-fKO (squares, n=6) murine brains, from age matched animals at day 6 to day 8 of life. Total RNA was subjected to DNase treatment and then used to generated cDNA. Thick horizontal central bars are mean, vertical bars are ± SD, and *p* value was calculated using Student's t-test.

Table S1: Gene Ontology analysis of upregulated genes in L2-fKO spleens using Fisher's t-test and FDR correction

Gene Ontology Biological Process	Total Genes	Observed	Expected	Fold Enrichment	p-value	False Discovery Rate
regulation of response to stimulus (GO:0048583)	3964	151	87.08	1.73	4.35E-12	3.42E-08
positive regulation of biological process (GO:0048518)	6356	213	139.62	1.53	2.73E-12	4.30E-08
positive regulation of response to stimulus (GO:0048584)	2297	102	50.46	2.02	1.42E-11	7.46E-08
cell surface receptor signaling pathway (GO:0007166)	1937	90	42.55	2.12	3.06E-11	9.63E-08
response to external stimulus (GO:0009605)	2425	105	53.27	1.97	2.67E-11	1.05E-07
external encapsulating structure organization (GO:0045229)	272	28	5.97	4.69	8.63E-11	1.70E-07
extracellular structure organization (GO:0043062)	271	28	5.95	4.7	7.98E-11	1.80E-07
extracellular matrix organization (GO:0030198)	270	28	5.93	4.72	7.38E-11	1.94E-07
response to stimulus (GO:0050896)	8591	259	188.71	1.37	2.14E-10	3.37E-07
immune response (GO:0006955)	1744	82	38.31	2.14	1.93E-10	3.37E-07

Table S2. Binding and non-binding motifs for ZFP36L2 based on gel shift assays

	Binding Motif	Transcript	species	Newly tested	Previously tested
1	GCUAUUUAUUC	*Ikzf2* ARE1	murine		Makita *et al.*, 2020 (27)
2	GAUAUUUAUUU	*Ikzf2* ARE2	murine		Makita *et al.*, 2020 (27)
3	CAUAUUUAUAA	*Ikzf2* ARE3	murine		Makita *et al.*, 2020 (27)
4	UCUAUUUAUUU	*Mpl*-WT	murine	1	
5	UAUAUUUAUUU	*Mpl*-Mut	murine	2	
6	AAUAUUUAUUU*[1]	*Irf8*	murine	3	
7	UUUAUUUAUUG	*Nfix*	murine	4	
8	AAUAUUUAUCU	*Lhr*-WT ARE1	murine		Ball *et al.*, 2014 (22)
9	AUUAUUUAUUU*[2]	*Lhr*-Mut ARE1	murine		Ball *et al.*, 2017 (12)
10	AUUAUUUAUUU*[2]	*Lhr* ARE2	human		Ball *et al.*, 2017 (12)
11	AAUAUUUAUGU	*Rest*	murine		Cargnin *et al.*, 2014 (35)
12	UUUAUUUAUUG*[3]	*Notch*	murine		Hodson *et al.*, 2010 (36)
13	UAUAUUUAUUC	*Ldlr*	human		Adachi *et al.*, 2014 (39)
14	GGUAUUUAUUU	*Elavl2* ARE4	murine	5	
15	AGUAUUUAUUU	*Elavl2* ARE5	murine	6	
16	UUUAUUUAUUG*[3]	*Elavl2* ARE6	murine	7	
17	CUUAUUUAUGA	*Star*	murine		Duan *et al.*, 2009 (40)
18	CUUAUUUAUUG	*Star*	murine		Duan *et al.*, 2009 (40)
19	AAUAUUUAUUU*[1]	*Apol11b*	murine	8	
	**Non-binding Motif**				
1	AUAAUUUAGAA	*Lhr*-WT ARE2	murine		Ball *et al.*, 2014 (22)
2	GAAAUUUAAAG	*Lhr*-WT ARE3	murine		Ball *et al.*, 2014 (22)
3	UGGAUUUAGGA	*Lhr*-WT ARE1	human		Ball *et al.*, 2017 (12)
4	GAAAUUUAAAA	*Lhr*-WT ARE3	human		Ball *et al.*, 2017 (12)
5	UAUAUUUAAAA	*Elavl2* ARE1	murine	9	
6	UGAAUUUACAG	*Elavl2* ARE2	murine	10	
7	UCCAUUUAUAU	*Elavl2* ARE3	murine	11	
8	CUGAUUUAAAG	*Fgf23*	murine	12	
